# How Emerging Digital Health Technologies Based on Dietary and Physical Activity Regulation Improve Metabolic Syndrome-Related Outcomes in Adolescents: A Systematic Review

**DOI:** 10.3390/metabo16020106

**Published:** 2026-02-02

**Authors:** Ruida Yu, Angkun Li, Yufei Qi, Jianhong Hu, Fei Peng, Shengrui Cao, Siyu Rong, Hao Zhang

**Affiliations:** 1 Department of Physical Education and Research, Central South University, Changsha 410083, China; 240912003@csu.edu.cn (R.Y.); yufeiqi@csu.edu.cn (Y.Q.); jianhonghu@csu.edu.cn (J.H.); csr777@csu.edu.cn (S.C.); 2 College of Public Health, Zhengzhou University, Zhengzhou 450001, China; 15036844766@163.com; 3 Department of Health and Physical Education, The Education University of Hong Kong, Taipo 999077, China; 4 College of Teacher Education, Capital Normal University, Beijing 100048, China; peng011227@163.com

**Keywords:** digital health, metabolic syndrome, combined intervention, adolescents, physical activity, dietary regulation

## Abstract

**Background:** Metabolic syndrome (MetS) is a pathological condition characterized by the co-occurrence of multiple metabolic abnormalities. The affected population is increasingly shifting toward younger age groups. Emerging digital health technologies, arising from advances in digital society, offer novel methodological tools for lifestyle-based interventions targeting metabolic risk. This systematic review aims to evaluate the effectiveness of emerging digital health technologies based on dietary and physical activity regulation in improving MetS-related outcomes among adolescents, including school-aged children. **Methods:** This review followed the PRISMA guidelines, systematically searched PubMed, Web of Science, Embase, MEDLINE, and Scopus, and screened eligible studies based on the PICO framework. **Results:** A total of 12 randomized controlled trials published between 2012 and 2025 were included in the analysis. Single device interventions (5/12) and dual device combinations (5/12) were the predominant approaches used in current digital health technology applications. Intervention content primarily focused on either physical activity alone (5/12) or combined exercise and nutrition interventions (7/12), with most programs lasting 3–6 months (7/12). Across the included digital health interventions, 13 MetS-related measures were assessed, including anthropometric/body composition measures (BMI, BMI z-score, WC, WHR, WHtR, and VFA), blood pressure measures (SBP/DBP), and biochemical markers (BG, HOMA-IR, TG, TC, HDL-C, and LDL-C). **Conclusions:** The available evidence supports the potential of digital health technologies to improve MetS-related outcomes. Although the selection of biochemical markers varied across studies, the findings highlight the importance of combined exercise and nutrition interventions or physical activity of moderate to high intensity in improving MetS. These results underscore the value of digital health technologies in elucidating the complex interactions among diet, physical activity, and metabolic responses. Overall, these findings support integrating digital health technologies into adolescent lifestyle interventions to facilitate more personalized monitoring and behavior support, and to potentially improve MetS-related outcomes. By promoting timely improvements in these outcome measures, such digital health interventions may have potential longer term implications for chronic disease prevention.

## 1. Introduction

Metabolic syndrome (MetS) is a pathological condition characterized by the co-occurrence of multiple metabolic abnormalities. MetS is widely recognized as an early indicator of cardiovascular disease, type 2 diabetes, and related complications [1,2]. The associated chronic diseases increase social and healthcare burdens and may reduce productivity and life expectancy in working-age populations, posing a major public health challenge. As a key early indicator of chronic diseases such as cardiovascular disease and type 2 diabetes, MetS is typically accompanied by central obesity [3], hypertension [4], impaired glucose metabolism [5], and dyslipidemia [6]. In clinical and epidemiological studies, MetS is not defined by a single measures; instead, it is identified through a comprehensive assessment of physiological and biochemical markers reflecting adiposity distribution, hemodynamic status, and glucose lipid homeostasis [7]. Among these markers, body mass index (BMI) and BMI z-score capture overall adiposity [8]. In contrast, waist circumference (WC), waist-to-hip ratio (WHR), waist-to-height ratio (WHtR), and visceral fat area (VFA) reflect visceral fat accumulation and the severity of central obesity [9]. Blood pressure (BP) reflects vascular load and autonomic regulation [10]. Fasting blood glucose (BG) and lipid indices, including triglycerides (TG), high-density lipoprotein cholesterol (HDL-C), low-density lipoprotein cholesterol (LDL-C), and total cholesterol (TC) are used to assess glucose and lipid metabolism [11]. These outcome measures underpin mainstream diagnostic criteria for MetS and are closely associated with metabolic pathway dysregulation [12], increased oxidative stress [13], and low grade chronic inflammation [14]. Collectively, they provide a systematic characterization of an individual’s metabolic risk profile [15].

In recent years, lifestyle changes have been accompanied by a rapid increase in the prevalence of MetS among adolescents [16], with both MetS and its associated abnormal outcome measuresshowing a clear shift toward younger age groups. Evidence indicates that overweight, central obesity, prehypertension, and abnormalities in glucose and lipid metabolism are no longer confined to middle-aged and older adults but are increasingly observed among adolescents, including school-aged children [17]. This trend is closely linked to changes in contemporary lifestyles and environmental contexts. From a behavioral perspective, adolescents commonly exhibit insufficient physical activity and prolonged sedentary behavior [18,19]. Academic pressure, increased screen time, and urban environmental constraints have contributed to a sustained decline in physical activity levels. Concurrently, adolescents often lack adequate nutritional knowledge regarding healthy dietary patterns and structure [20]. These behavioral characteristics collectively contribute to increased consumption of high-calorie foods, refined carbohydrates, and trans fats, alongside insufficient intake of whole grains, fruits, vegetables, and high-quality fats. Collectively, these factors result in an imbalance between energy intake and expenditure. By promoting insulin resistance and dysregulated lipid metabolism [21], these behaviors accelerate the accumulation of metabolic risk factors [22].

During adolescence, the body undergoes a critical period of metabolic system plasticity. Metabolic dysregulation induced by physical inactivity or unhealthy dietary habits during this stage may persist into adulthood via a “metabolic memory effect” [23], thereby increasing the risk of chronic diseases, including cardiovascular disease and type 2 diabetes [24]. Therefore, once the relevant outcome measures of metabolic syndrome become abnormal, timely guidance and intervention are required to support normal metabolic function and development in adolescents [25]. Although traditional lifestyle intervention strategies (e.g., health education, exercise prescriptions, and dietary guidance) have demonstrated efficacy in improving MetS-related outcome measures [26], they remain largely dependent on face-to-face delivery. Such approaches have limited reach, make it difficult to standardize intervention intensity, and lack continuous monitoring and real-time feedback during individual behavior change. Consequently, intervention adherence tends to decline, thereby limiting the sustainability of health improvements [27].

With advances in digital society and technology, digital health technologies have introduced novel methodological tools for lifestyle-based metabolic-related outcome measures interventions [28], offering unprecedented opportunities to implement interventions among adolescents across diverse settings, including everyday living and learning environments. Emerging digital health technologies, exemplified by wearable devices [29], mobile health applications [30], telemedicine [31], and artificial intelligence [32], have been extensively validated for their precision, adherence, and sustainability in the clinical prevention and management of MetS. The strength of digital health technologies lies in their ability to integrate behavioral monitoring, real-time feedback, and personalized adaptations into dynamic intervention systems, thereby enabling sustained improvements in physical activity and dietary behaviors.

Despite increasing academic interest in applying digital health technologies to MetS-related outcome measures and lifestyle interventions among adolescents, substantial heterogeneity exists in technology types, intervention content, and intervention duration. Furthermore, inconsistent selection of outcome measures limits the ability to conduct comprehensive, multi-measures assessments of MetS. A systematic synthesis of the effects of digital health technologies on MetS-related outcome measures remains lacking, thereby constraining a clear understanding of their specific role in metabolic risk management. Moreover, previous systematic reviews in this field have predominantly focused on specific populations, including older adults [33], individuals with diabetes [34], cardiovascular disease [35], mental disorders [36], and pediatric cancer survivors [37]. To date, there remains a lack of comprehensive and systematic analyses specifically addressing physical activity and dietary patterns among adolescents.

Accordingly, this systematic review addresses the following core question: “How do digital health technologies based on dietary and physical activity regulation improve MetS-related outcomes among adolescents, including school-aged children?” This review systematically synthesizes evidence from randomized controlled trials to evaluate how different digital health technologies regulate physical activity and dietary behaviors and influence MetS-related measures, including BMI, BMI z-score, WC, WHR, BP, BG, and lipid profiles. Ultimately, this review aims to provide evidence-based guidance and practical implementation pathways for the prevention of early-onset metabolic syndrome in adolescent populations.

## 2. Materials and Methods

This systematic review was conducted in accordance with the Preferred Reporting Items for Systematic Reviews and Meta-Analyses (PRISMA) guidelines [38]. The review protocol was prospectively registered in the PROSPERO database (CRD420251265901).

### 2.1. Search Strategy

**Figure 1:**
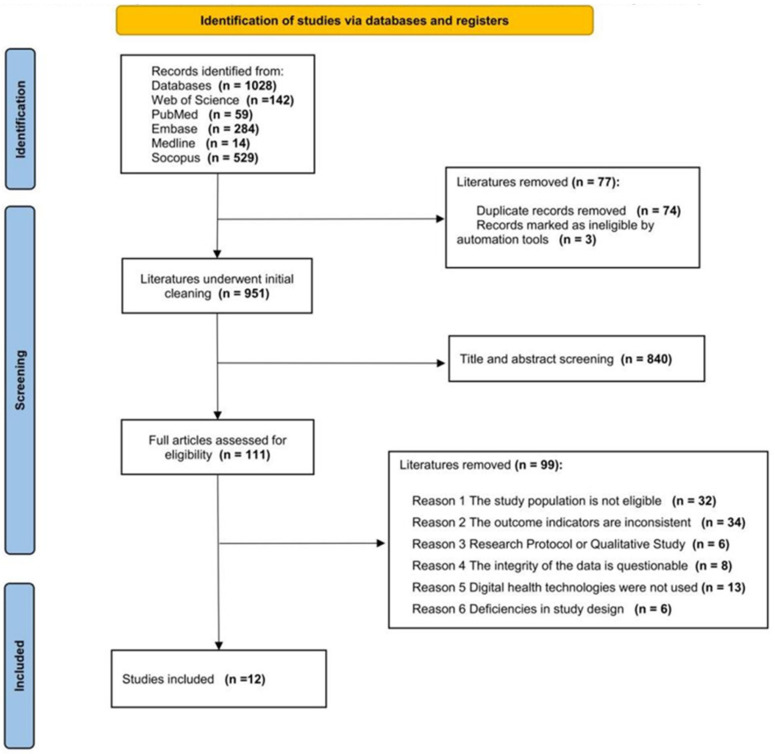
PRISMA Flow Diagram for Study Search and Screening.

On 10 October 2025, we conducted a comprehensive search of PubMed, Web of Science, Embase, MEDLINE, and Scopus. Peer-reviewed articles published in English were eligible for inclusion, with no restrictions on publication year. We excluded reviews, meta-analyses, and grey literature (e.g., commentaries, guidelines, editorials, letters to the editor, conference abstracts, working papers, and policy papers), as well as non-randomized or uncontrolled studies. The complete search strategy is detailed in the Supplementary Materials File S1 and Table S1–S4. All retrieved records were exported to spreadsheets, and duplicate entries were removed. The study selection process is summarized in Figure 1.

### 2.2. Eligibility Criteria

We included only original, peer-reviewed articles published in English. We excluded reviews, meta-analyses, and grey literature (e.g., commentaries, guidelines, editorials, letters to the editor, conference abstracts, working papers, and policy papers), as well as non-randomized or uncontrolled studies. The detailed inclusion and exclusion criteria are summarized in Table 1.

### 2.3. Data Collection and Screening Process

All retrieved records were extracted into a Microsoft Excel spreadsheet using a standardized data extraction format. Key information extracted from each included study comprised titles, authors, publication year, journal, study design (randomized controlled trial), data sources, intervention characteristics, sample size, and outcome measures.

Based on the predefined eligibility criteria, two independent reviewers (H.Z. and R.D.Y.) screened titles and abstracts. Following the initial screening, the full texts of potentially eligible articles were assessed for final inclusion. Any disagreements were resolved through consultation with a third reviewer (Y.F.Q.). Data extraction was independently performed by two reviewers (H.Z. and R.D.Y.) using standardized extraction forms. The primary statistical methods used to assess changes in outcome measures included tests of normality and homogeneity of variance (e.g., Shapiro–Wilk, Kolmogorov–Smirnov, and Levene tests); between-group comparisons (e.g., independent-samples *t*-tests, chi-square tests, and Wilcoxon signed-rank tests); repeated-measures and group × time interaction analyses (e.g., repeated-measures ANOVA and two-factor mixed-design MANOVA/ANOVA); analysis of covariance (ANCOVA) to adjust for covariates (e.g., maturity, sex, and intervention type); and corrections for multiple comparisons (e.g., Bonferroni correction).

Due to several methodological limitations, a quantitative meta-analysis was not performed. First, most included studies did not provide access to raw data, precluding reanalysis or data pooling. Second, MetS was assessed using heterogeneous outcome measures spanning multiple dimensions (e.g., BP and central obesity), rather than a single quantitative measures. In addition, heterogeneity in sample characteristics, reporting formats, and statistical methods further limited comparability across trials [39].

### 2.4. Risk of Bias Assessment

Two independent reviewers (H.Z. and R.D.Y.) independently conducted the risk of bias assessment. Any disagreements were resolved through consultation with a third reviewer (Y.F.Q.). The risk of bias of included studies was assessed using the Cochrane Risk of Bias tool for randomized trials (RoB 2.0). The RoB 2.0 tool evaluates potential sources of bias in randomized controlled trials across five domains. These domains include: (1) bias arising from the randomization process; (2) bias due to deviations from intended interventions; (3) bias due to missing outcome data; (4) bias in measurement of the outcome; and (5) bias in selection of the reported result. Each domain was judged as having low risk, some concerns, or high risk of bias.

## 3. Results

### 3.1. Study Selection

The systematic search of PubMed, Web of Science, Embase, MEDLINE, and Scopus yielded 1028 records. After removal of 74 duplicates and 3 records deemed ineligible, 951 records remained. Title and abstract screening of the 951 records identified 111 articles for full-text assessment. Of the 111 full-text articles assessed for eligibility, 99 were excluded for the following reasons: (1) ineligible population (non-adolescent cohort) (*n* = 32); (2) outcomes not aligned with MetS assessment (e.g., no reference to MetS diagnostic criteria) (*n* = 34); (3) ineligible study design (non-randomized, uncontrolled, or qualitative research) (*n* = 6); (4) questionable data integrity (data authenticity could not be verified and additional information could not be obtained from authors) (*n* = 8); (5) no use of digital health technologies (*n* = 13); and (6) major methodological flaws (e.g., unclear control group or protocol deficiencies) (*n* = 6). Ultimately, 12 studies met the inclusion criteria and were included in this systematic review.

### 3.2. Study Characteristics

The key characteristics included in the study and the associated risk assessments are summarized in Supplementary Materials. The included trials were published between 2012 and 2025: one in 2012 [40], one each in 2016, 2017, 2021, and 2023 [41,42,43,44], five in 2024 [45,46,47,48,49], and two in 2025 [50,51]. The studies were conducted across diverse geographic regions. The majority of studies were conducted in the United States (*n* = 6) [41,42,43,44,45,49]. The remaining studies were conducted in Spain (*n* = 2) [46], Portugal (*n* = 1) [51], China (*n* = 1) [48], Australia (*n* = 1) [40], and Egypt (*n* = 1) [50]. Overall, the studies were distributed across North America (United States), Europe (Spain and Portugal), Asia (China), Oceania (Australia), and Africa (Egypt). Sample sizes ranged from 18 participants [48] to 430 participants [46], totaling 1633 participants across all included studies. Study populations included generally healthy adolescents, adolescents with overweight or obesity, those with prolonged sedentary behavior, and those at elevated risk of cardiovascular disease.

Most studies enrolled school-aged children or adolescents with overweight or obesity, whereas a minority included college students or adolescents at elevated cardiovascular risk. Specifically, eight studies enrolled school-aged children [40,42,43,46,47,49,50,51], two targeted college students [41,48], and two included both age groups [44,45]. Mean participant age ranged from 13.18 years [40] to 19.7 years [41]. Intervention duration ranged from 8 weeks [48,50] to 18 months [44]. Interventions were delivered primarily in school, community, or clinical settings, highlighting the potential for digital health technologies to be implemented across diverse real-world contexts.

### 3.3. The Regulation of Physical Activity and Diet by Different Digital Health Technologies

Across the 12 included studies, digital health interventions varied in (1) technology modality, (2) intervention content, and (3) protocol design. This heterogeneity may limit comparability across studies.

Regarding technology modality, the included studies were highly heterogeneous. Overall, interventions were categorized as single device (5/12) [41,43,46,47,50], dual device (5/12) [40,44,45,48,51], or multi-component hybrid approaches (2/12) [42,49]. Step-tracking applications were the most common single technology component. Wearable devices (e.g., Fitbit), email, and virtual reality (VR) devices were also used as standalone tools in some trials. Several trials combined core technologies with additional components such as remote video visits [44], text message support [40], and social network interventions [51] to target physical activity and dietary behaviors. In more integrated school or clinical settings, multi-component hybrid interventions incorporated elements such as electronic health records, online educational modules, and cardiovascular health visualization tools [42,49]. Step-tracking apps and wearables (e.g., Fitbit) constituted the core technologies used across studies. These technologies were used either alone or combined with additional modules. Other trials extended these approaches by incorporating additional modalities to address different implementation contexts and intervention needs. Some trials combined telemedicine modalities (e.g., remote video visits and private social network-based group CBT) with self-monitoring to support changes in physical activity and diet. Kepper et al. implemented the digital counseling tool PREVENT in a clinical setting [49]. Abd El-Khalek et al. incorporated VR games within a gamified intervention to enhance exercise engagement [50]. Overall, mHealth and wearable technologies were most frequently used, complemented by clinical digital tools and immersive technologies, with several interventions integrated into school or healthcare settings (Table 2).

Regarding intervention content, studies were categorized as physical activity–only interventions (5/12) or combined exercise–nutrition interventions (7/12). Five trials targeted physical activity alone, primarily using step-tracking apps and wearable devices. In some trials, participants were encouraged to increase activity levels through progressively increasing step goals [46,47]. Beyond step counting, Sun et al. incorporated additional training modalities, such as high-intensity interval training (HIIT) [48]. Specific components in this category included step-goal setting [45] and self-monitoring [43]. The most frequently used approach was the combined exercise–nutrition intervention, which typically paired dietary guidance with physical activity monitoring. In these combined interventions, physical activity components were often emphasized, whereas dietary components were commonly delivered via personalized guidance or self-monitoring. Abd El-Khalek et al. augmented the combined intervention by integrating aerobic exercise with VR exercise games alongside dietary guidance [50]. None of the 12 studies evaluated dietary regulation as a standalone intervention, suggesting that dietary components were typically used as adjuncts rather than primary digital health intervention targets. The earliest dietary component was delivered through nutrition workshops [40], with later studies shifting toward personalized guidance supported by digital technologies. Later studies shifted toward digitally supported, personalized guidance [44] (Table 2).

Regarding protocol design, interventions were categorized as short-term (8–10 weeks) [46,48,50], medium-term (3–6 months) [41,42,43,45,47,49,51], or long-term (≥12 months) [40,44]. Most trials implemented medium-term protocols (3–6 months; 7/12), whereas fewer used short-term (3/12) or long-term (2/12) protocols. Short-term protocols were more common in physical activity-focused programs and may be suitable for initiating behavior change (e.g., increasing daily activity). Medium-term protocols frequently combined wearables with telemedicine and/or social platforms and provided a practical observation window for behavior change and improvements in metabolic measures. Long-term protocols also used combined approaches and often incorporated enhanced dietary strategies and nutrition workshops, which may support more sustained improvements in health behaviors and metabolic outcome measures (Table 2).

### 3.4. The Impact of Digital Health Technology Interventions on MetS-Related Outcomes

Across the 12 included studies, the primary MetS-related outcomes assessed in digital health interventions included BMI, BMI z-score, WC, WHtR, WHR, VFA, BP, BG, HOMA-IR, TG, TC, HDL-C, and LDL-C. These outcome measures are commonly used in pediatric and adolescent definitions and assessments of MetS. BMI and BMI z-scores are widely used screening measures for assessing overall obesity levels, providing an indirect reflection of metabolic disorder risk among adolescent populations [52,53,54]. WC and related indices (e.g., WHR) are markers of central adiposity [55], and are included in diagnostic criteria for MetS by organizations such as the International Diabetes Federation (IDF) [56]. BP and BG are routinely assessed in this context and can indicate alterations related to insulin resistance and impaired glucose metabolism [57,58]. Accordingly, these measures provide complementary information on cardiometabolic risk profiles in adolescents [59]. MetS is commonly accompanied by dyslipidemia [60], which is assessed using lipid measures such as TG and cholesterol fractions [61]. Collectively, these outcome measures were reported across the 12 studies and were used to evaluate changes in MetS-related outcome measures following digital health technology interventions (Table 3 and Table 4).

Across all 12 studies, BMI or BMI z-score was included as a primary anthropometric outcome. Most trials prioritized BMI/BMI z-score, and five reported significant reductions in at least one of these measures [42,44,46,50,51]. For Gómez-Cuesta et al., they demonstrated a statistically significant increase in BMI values within the control group (*p* = 0.001), whereas no significant change was observed in the intervention group over 10 weeks [46]. In Ptomey et al., the combined intervention resulted in a significantly greater reduction in BMI than the control group (*p* = 0.03) [44]. Similarly, Chen et al. reported significantly greater reductions in both BMI and BMI z-score in the intervention group compared with the control group (*p* = 0.001 for both) [42]. In Abd El-Khalek et al., BMI decreased significantly within both groups (within-group *p* < 0.001), with a significantly greater reduction in the intervention group (between-group *p* = 0.001) [50]. Although Ramalho et al. observed significant reductions in BMI z-scores over time in both groups (*p* = 0.006), no significant group-by-time interaction was detected [51]. Furthermore, some studies reported no significant changes or merely showed a downward trend.

Central adiposity is a key component of MetS assessment and reflects body fat distribution and visceral fat accumulation. Eight studies assessed central adiposity outcomes, including WC, WHtR, WHR, and VFA [41,42,43,44,46,47,48,50]. Two studies reported significant reductions in WC [44,50], two reported significant improvements in WHR [48,50], and one reported a significant reduction in VFA [48]. Ptomey et al. reported a significantly greater reduction in WC in the intervention group compared with the control group (*p* = 0.002) [44]. Sun et al. found a significant reduction in WHR in the intervention group (*p* = 0.033), while VFA decreased significantly in both the intervention and control groups (*p* = 0.001 and *p* = 0.003, respectively) [48]. For Abd El-Khalek et al., WC and WHR improved significantly within both groups (within-group *p* < 0.001), but between-group differences were not significant [50]. Multiple other studies failed to observe significant inter-group differences in central obesity measures.

BP is commonly assessed as an early cardiometabolic risk marker. Five of the 12 studies reported improvements in BP outcomes [41,42,45,48,49]. Specifically, two studies reported significant reductions in both systolic blood pressure (SBP) and diastolic blood pressure (DBP) [48,49]; one reported a significant reduction in DBP [42]; and one reported a significant reduction in SBP [45]. Sun et al. observed significant decreases in both SBP and DBP within the intervention group (*p* = 0.018 and *p* = 0.008, respectively) [48]. Chen et al. reported that overall blood pressure decreased significantly in the intervention group compared with the control group (*p* = 0.001), although SBP alone showed no significant difference [42]. Kepper et al. reported a significant reduction in SBP in the intervention group (within-group *p* = 0.009; between-group *p* = 0.001), while DBP decreased significantly within the intervention group (within-group *p* = 0.009) but not between groups [49]. By contrast, Schweitzer et al. reported no significant between-group differences in SBP/DBP [41], and Bicki et al. observed a non-significant between-group difference despite a −2.3 mmHg change in SBP (95% CI, −6.5 to 1.8) over 6 months [45].

Only two of the 12 studies measured BG [48,49], and neither reported statistically significant changes. Although Sun et al. reported no significant change in BG, HOMA-IR showed a downward trend [48]. Kepper et al. also reported a downward trend in BG; however, due to substantial loss to follow-up, no significance testing was performed [49].

BL outcomes were measured in two of the 12 studies [48,49]. In Sun et al., TG decreased significantly in the intervention group (*p* = 0.004), whereas TC, HDL-C, and LDL-C showed no significant changes; BG showed no significant between-group difference and HOMA-IR exhibited a non-significant downward trend. In Kepper et al., downward trends were reported for BG and TC; however, statistical significance testing was not performed due to high missingness.

### 3.5. The Impact of Differently Designed Digital Health Technology Interventions on MetS-Related Outcomes

Across the 12 studies, intervention duration was categorized as short term (8–10 weeks) [46,48,50], medium-term (3–6 months) [41,42,43,45,47,49,51], or long-term (≥12 months) [40,44]. Most studies used medium-term interventions (3–6 months; 7/12), whereas fewer used short-term (3/12) or long-term (2/12) interventions. Except for long-term interventions, short- and medium-term protocols targeted either physical activity alone or combined exercise and nutrition (Table 5).

Among the three short-term studies (8–10 weeks), two used physical activity-only interventions and reported improvements in overall and/or central adiposity. These studies reported reductions in WHR and WHtR in intervention groups, but between-group differences were not statistically significant [46,48]. One combined exercise–nutrition study reported that adding VR gaming to dietary guidance and aerobic exercise produced a greater reduction in BMI than the same program without VR (between-group *p* = 0.001) [50]. Sun et al. reported that higher exercise intensity was associated with a significant within-group reduction in VFA (*p* = 0.001) after 8 weeks, along with decreases in SBP (*p* = 0.018) and DBP (*p* = 0.008) and a reduction in triglycerides (*p* = 0.004). However, blood glucose did not change significantly, and insulin resistance indices showed only nonsignificant downward trends [48]. Similar changes in physiological and biochemical measures were not observed in the short-term interventions by Abd El-Khalek et al. and Gómez-Cuesta et al., which did not include higher-intensity training components.

Among the seven medium-term studies (3–6 months), most used combined exercise and nutrition interventions (4/7). Physical activity only interventions were less common in this timeframe and generally yielded modest effects. Among physical activity-only interventions, Bowen-Jallow et al., Bicki et al., and Mateo-Orcajada et al. targeted step counts. Bowen-Jallow et al. reported no significant between-group differences in BMI or WC after the intervention (*p* = 1.00 and *p* = 0.83, respectively) [43]. Bicki et al. reported a nonsignificant between-group difference in SBP (−2.3 mmHg; 95% CI, −6.5 to 1.8; *p* = 0.12) [45]. Mateo-Orcajada et al. reported no significant between-group differences in BMI or WC (*p* = 0.09 and *p* = 0.61), but WHR improved significantly in the intervention group compared with the control group (*p* = 0.02) [47]. Compared with physical activity-only interventions, Chen et al. reported significant between-group differences in BMI, BMI z score, and BP after a 6-month combined intervention that integrated dietary guidance with physical activity monitoring (all *p* = 0.001) [42]. Ramalho et al. reported significant within-group reductions in BMI z scores over 6 months (*p* = 0.006), but no significant group × time interaction [51]. In a 3-month clinical intervention using the PREVENT counseling tool, Kepper et al. reported downward trends in BMI z score, TC, and BG across groups. SBP and DBP decreased significantly within the intervention group (both *p* = 0.009) [49]. In contrast, Schweitzer et al.’s 24-week combined intervention did not yield significant between-group differences in BMI, WC, WHR, SBP, or DBP (*p* = 0.80, 0.41, 0.21, 0.92, and 0.80, respectively) [41].

Two long-term studies (≥12 months) used combined exercise and nutrition interventions, but intervention content differed. Lubans et al. delivered nutrition workshops, lunchtime activities, and self-monitoring and reported only nonsignificant downward trends in BMI and BMI z score [40]. By contrast, Ptomey et al. implemented an 18-month program (6 months of weight reduction followed by 12 months of maintenance) that incorporated an enhanced traffic-light diet and physical activity monitoring. They reported significant within-group reductions in BMI and WC in the intervention group (*p* = 0.03 and *p* = 0.002, respectively) [44].

## 4. Discussion

### 4.1. Key Findings on Digital Health Technologies for Regulating MetS-Related Outcomes

This review, including 12 randomized controlled trials, suggests that emerging digital health technologies may improve 13 MetS-related measures in school-aged children and adolescents. Across included studies, outcomes encompassed anthropometric measures, central adiposity indices, and blood-based physiological and biochemical markers. For anthropometric outcomes, five trials reported significant improvements in BMI and/or BMI z score following digital health interventions. hese interventions ranged from single-component physical activity programs to combined exercise–nutrition programs and included short-, medium-, and long-term durations. This pattern suggests that digital health interventions offer flexibility in protocol design for anthropometric measures such as BMI and BMI z score, consistent with Lister et al. [62]. This may be because anthropometric measures are relatively stable and easy to obtain compared with other markers [63,64], and adolescence is a sensitive period for changes in adiposity-related measures [65]. Consequently, statistically significant changes in BMI and BMI z score may be easier to detect within typical trial designs. Future studies should extend follow-up while maintaining intervention intensity to determine whether improvements in anthropometric measures translate into sustained reductions in central adiposity and improvements in physiological/biochemical markers.

For central adiposity outcomes, five studies reported significant improvements in WC, WHR, WHtR, and VFA. However, only two studies reported significant changes in WC, and more than half of the included trials found no significant between-group differences. WHR and WHtR are derived from WC, whereas VFA is assessed separately. Moreover, increases in visceral fat often correspond to greater WC [66], underscoring WC as a key measures of MetS-related risk. WC is important not only as a marker of abdominal obesity [67] but also because greater visceral adiposity is linked to insulin resistance [68], chronic low-grade inflammation [69] and dysregulated lipid metabolism [70]. These processes may contribute to the clustering of abnormalities such as hyperglycemia, dyslipidemia, and elevated blood pressure [71]. Accordingly, changes in WC are often viewed as an outcome measures of whether digital health interventions affect central adiposity within the MetS pathway. Therefore, the limited improvements in WC suggest that, with current evidence, digital health interventions may show earlier effects on overall anthropometric measures (e.g., body weight) than on central adiposity. Achieving sustained reductions in visceral fat and abdominal obesity may require longer interventions and/or higher-intensity physical activity. Several factors may contribute to this pattern. First, inconsistent breathing during measurement may introduce random error [72,73]. Second, during growth spurts, increases in height and changes in fat distribution may attenuate absolute changes in WC, making improvements harder to detect [74]. Third, some studies may have delivered insufficient intervention intensity/dose or used active control conditions (e.g., routine physical activity and health education). This may have reduced between-group behavioral contrasts, thereby attenuating observable effects on WC [75]. Taken together, these considerations suggest that improvements in central adiposity may require longer interventions and/or moderate-to-high-intensity exercise. Consistent with this interpretation, Rajjo et al. reported that longer, higher-intensity exercise interventions may be more beneficial for improving central adiposity [76]. Future studies should extend follow-up, better standardize and monitor exercise dose, and report central adiposity indices (e.g., WHR and VFA) where feasible. This approach would enable a more accurate assessment of the effects of digital health interventions on central adiposity.

For blood-based outcomes, three studies reported significant improvements in blood pressure, one reported significant improvements in blood glucose, and one reported significant improvements in lipid outcomes following digital health interventions. These findings suggest that blood pressure may be more responsive, whereas evidence for improvements in blood glucose and lipids remains limited. Some studies reported reductions in both systolic and diastolic blood pressure or in one component, suggesting that digital health interventions may affect blood pressure by increasing physical activity, improving cardiorespiratory fitness, and supporting vascular regulation. By contrast, evidence for blood glucose and lipid outcomes was less consistent. Only a few studies reported decreasing trends in insulin resistance-related markers or significant reductions in triglycerides, whereas most blood glucose and other lipid parameters did not change significantly. This may reflect that blood glucose and lipid parameters change relatively slowly and are influenced by multiple factors, including baseline metabolic status, hormonal fluctuations, and energy intake [77,78]. In addition, infrequent measurement, variable sampling conditions, and heterogeneous laboratory protocols may reduce sensitivity to change; detectable improvements may therefore require longer interventions and follow-up [79,80]. Among lipid parameters, TG, TC, HDL-C, and LDL-C appeared more responsive to higher-intensity physical activity than to lower-intensity combined programs, with significant changes reported more often in the former. This pattern suggests sensitivity to exercise intensity [81]. Future studies should better prescribe and monitor exercise intensity and dose, strengthen continuous monitoring and support for dietary behaviors, and extend intervention duration to more accurately evaluate effects on blood glucose and lipid outcomes.

This review found that digital health interventions primarily relied on step-tracking apps and Fitbit wearables and could be categorized as single device (5/12), dual device (5/12), or multi-component hybrid approaches (2/12). This pattern may reflect the combined influence of device characteristics, implementation contexts, and feasibility considerations. From a technical perspective, compared with immersive modalities such as VR and AR, step-tracking apps and Fitbit wearables offer simpler operation, lower cost, and intuitive data visualization. These features may better align with adolescents’ typical digital literacy and usability preferences. In addition, their applicability across school, home, and clinical settings, together with real-time monitoring and feedback, may partially mitigate limitations of traditional interventions, such as difficulty quantifying physical activity and delays in feedback delivery [82]. However, across the 12 studies, most digital health approaches relied primarily on monitoring, feedback, and guidance. In contrast, Abd El-Khalek et al. incorporated VR-based games to increase engagement in adolescent interventions [50]. Moreover, most interventions prioritized promoting physical activity. Even in combined exercise–nutrition interventions, dietary components were often delivered via conventional digital channels (e.g., online videos, web-based courses, and social media) rather than through continuous dietary monitoring. As digital technologies evolve, adolescents may increasingly prefer more engaging modalities [83], and real-time feedback on dietary behaviors may represent an important unmet need. Based on these findings, future research could explore incorporating immersive, motion-sensing modalities (VR, AR, and extended reality [XR]) alongside newer digital health tools (e.g., smartwatches and wearable glasses) that may enable real-time dietary behavior logging and feedback, provided feasibility and safety are rigorously evaluated. In summary, future work could prioritize more continuous monitoring and feedback for dietary behaviors and evaluate multi-technology approaches over appropriate intervention durations to improve MetS-related outcomes in adolescents.

### 4.2. Mechanisms of Digital Health Technologies in Combined Exercise and Dietary Interventions

Among the 12 randomized controlled trials included in this review, seven implemented combined exercise–nutrition interventions, whereas five focused on physical activity alone. No trials evaluated dietary interventions as a standalone strategy. This pattern suggests that, within adolescent digital health research, combined interventions are more commonly adopted to address MetS-related measures. This may reflect the challenge of sustaining dietary change in real-world settings, where eating behaviors are strongly shaped by environmental and social influences [84]. In contrast, digitally supported physical activity promotion may produce more readily observable short-term changes. Hodder et al. reported that exercise interventions typically increase moderate-to-vigorous physical activity but show limited evidence for sustained dietary improvement [85]. This suggests that increasing physical activity may be more feasible than sustaining dietary change in real-world settings, consistent with conclusions drawn by Li et al. [86]. Accordingly, no standalone dietary intervention trials were identified among the included studies. Compared with physical activity-only programs, combined interventions used more diverse technology configurations. Examples included enhanced traffic light dietary guidance paired with activity monitoring, dietary counseling integrated with online self-tracking, nutrition consultations combined with VR-based aerobic exercise, and electronic health record-enabled cardiovascular health visualization linked to dietary guidance. Overall, core technologies still centered on step-tracking apps and Fitbit wearables, with dietary components commonly delivered as personalized guidance [87].

The primary reason why combined interventions are favoured by most studies likely lies in their greater alignment with the underlying logic that metabolic syndrome stems from long-term imbalances between energy intake and expenditure, leading to metabolic abnormalities [21]. Digital health technologies that integrate exercise and nutrition may help address this challenge. Energy balance is a key target for precision lifestyle interventions. Compared with traditional approaches, digital health technologies can use wearables to monitor physical activity (energy expenditure) in real time and deliver dietary guidance to support management of energy intake. This approach may help mitigate metabolic compensation associated with binge eating or excessive dietary restriction [88,89], thereby attenuating adverse metabolic consequences of energy imbalance. Advances in digital technology have enabled more integrated monitoring and feedback, which may strengthen behavioral engagement and relevance. Many combined interventions integrate dietary and physical activity data through applications. Ramalho et al. used an online self-monitoring system integrating physical activity and dietary records, thereby shifting behavior-change processes toward the individual level. This approach may enhance motivation and support behavior change through self-monitoring and self-regulation [51]. In addition, supplementary features such as text reminders, online courses, and social support can provide additional resources for dietary planning and physical activity implementation. These features may partially reduce the gap between dietary and physical activity guidance and lower implementation barriers that are common in traditional combined interventions. Intervention duration may also influence the magnitude of observed effects. A medium-term intervention (3–6 months) may provide sufficient time for coordinated development of dietary and physical activity behaviors, consistent with findings from a meta-analysis of weight loss among adults with type 2 diabetes [90]. Moschonis et al. also reported that longer-term interventions (12–18 months) with sustained digital support may help consolidate healthy habits and reduce short-term relapse [91]. Notably, much of the supporting evidence is derived from different populations and study designs. Therefore, these inferences require validation in adolescent populations with MetS using appropriately designed studies.

Although both physical activity-only and combined exercise–nutrition interventions are mechanistically plausible, some outcomes did not reach statistical significance. Comparing design and implementation across studies suggests several contributors to null findings, including inadequate control of confounding, suboptimal study design, and insufficient exercise intensity. First, small sample sizes, high dropout rates, and missing outcome data likely reduced statistical power. In some studies, this precluded formal hypothesis testing and may have obscured modest effects. Kepper et al. reported that analyses of BG and TC were precluded by limited sample size and high dropout [49]. Bowen-Jallow et al. noted that small sample size, unequal allocation ratios, and dropout limited the ability to detect between-group differences in BMI and WC [43]. Ramalho et al. reported that missing data precluded more complex models, limiting analyses to within-group changes in BMI z scores [51]. Second, control groups often received routine physical activity or health education; in some studies, shared intervention exposure may have diluted between-group differences. In Schweitzer et al., the control group received feedback and goal-setting prompts after baseline dietary and physical activity assessments. Inadequate adjustment for potential confounding may have contributed to nonsignificant between-group differences in BMI, WC, WHR, and BP [41]. Similarly, in Kepper et al., control participants received routine care that included counseling on physical activity and nutrition. This may have reduced the contrast between groups, contributing to nonsignificant differences in BMI z score [49]. Third, intervention dose may have been insufficient, particularly with respect to exercise intensity, program content, and dietary guidance. This may have limited the extent of physiological change achievable within the study period. For physical activity–only interventions, exercise intensity and program content are likely critical determinants of effect. Sun et al. reported improvements across multiple outcomes (e.g., WHR, VFA, BP, and TG) after an 8-week physical activity-only intervention that incorporated HIIT [48]. Bicki et al. reported no significant between-group improvement in SBP after a 6-month pedometer intervention. This may reflect limited personalization and insufficient engagement to motivate changes in activity patterns [45]. Gómez-Cuesta et al. reported that a short-term intervention using step-tracking apps improved BMI [46]. This contrasts with Bowen-Jallow et al. and Mateo-Orcajada et al., who relied primarily on step tracking and reported limited effects despite longer intervention durations [43,47]. This difference may reflect clearer daily activity targets in Gómez-Cuesta et al., lower adherence in Bowen-Jallow et al., and limited control of activity frequency/intensity in Mateo-Orcajada et al. These findings suggest that physical activity-only interventions may require sufficient intensity to produce measurable effects. For interventions lasting >3 months, engaging and enjoyable content may be needed to support adolescent motivation and adherence. Exercise intensity may also be important in combined interventions. For example, Abd El-Khalek et al. reported within-group improvements in central adiposity measures in a short-term program combining aerobic exercise with VR games [50]. This contrasts with Schweitzer et al. and Chen et al. [41,42], possibly because interventions relying mainly on online guidance may have delivered lower exercise intensity than supervised aerobic exercise combined with VR games. To better estimate intervention effects, future studies should define and quantify exercise dose, minimize contamination in control groups, and use objective tools for continuous behavioral monitoring. This approach would clarify the effects of digital health interventions across MetS-related outcome domains.

These mechanistic considerations provide potential implications for clinical and public health practice. Future interventions for adolescents with MetS could adopt a combined framework centered on physical activity monitoring and supplemented by dietary guidance. Priority could be given to platforms capable of monitoring and providing feedback on both dietary intake and energy expenditure, thereby enhancing coordination between intervention components. Intervention duration could be planned around a 3–6-month cycle, with follow-up as appropriate. For subgroups with chronic conditions (e.g., obesity or cardiovascular disease), longer intervention periods may be considered to support maintenance of behavior change. In summary, digital health-enabled combined interventions may improve MetS-related outcomes by targeting mechanisms such as energy balance, offering a promising avenue for the prevention of MetS in adolescents. Future research should optimize exercise intensity, intervention duration, and alignment between technology features and individual behavior to improve the effectiveness of digital health interventions for MetS-related outcomes in adolescents.

### 4.3. Advantages and Limitations

This review has three main strengths: population focus, analytical framework, and outcome selection. (1) Population focus: To inform interventions for the increasingly younger onset of MetS, we focused on school-aged children and adolescents aged 8–22 years. Compared with prior reviews that focused primarily on older adults or clinical populations (e.g., individuals with diabetes), this review synthesizes evidence on digital health technologies targeting diet and physical activity in adolescents, Provide evidence to guide the prevention of MetS. (2) Analytical framework: We summarized the statistical methods used to evaluate outcome changes, which improves transparency in interpreting findings. In addition, we organized findings across three dimensions: technology modality (single device, dual device, multi-component hybrid), intervention content (physical activity–only vs. combined exercise–nutrition), and duration (short-, medium-, and long-term). This structure may inform technology selection and program design in practical settings. (3) Outcome selection: We emphasized commonly used MetS-related outcome measures, including overall adiposity (BMI/BMI z-score), central adiposity (WC and WHR), and cardiometabolic markers (BP, BG, and lipid measures). By mapping outcomes to intervention types, this synthesis helps identify which targets are most frequently addressed by different technologies and may guide optimization of future intervention protocols.

This review has several limitations. (1) Population and sample size: Most included studies enrolled adolescents with overweight or obesity, and sample sizes varied substantially (18–430 participants). The limited statistical power of small-sample studies (e.g., *n* = 18) may have reduced the precision and stability of some estimates. (2) Analytical heterogeneity: The included studies used heterogeneous statistical approaches (e.g., independent-samples *t*-tests and repeated-measures ANOVA). In addition, raw data were unavailable for several studies, precluding a quantitative meta-analysis and limiting this work to narrative synthesis. As a result, overall effect sizes and consistency across trials could not be formally quantified. (3) Risk of bias and blinding: All included trials required wearable-based monitoring for physical activity components. This design makes blinding participants and personnel challenging and may introduce performance and detection bias in outcome assessment. (4) Publication bias: Our exclusion of grey literature (e.g., conference abstracts, dissertations, and trial registries) were not captured, which may have influenced our overall conclusions.

Future studies could conduct multicenter, adequately powered randomized controlled trials to improve representativeness across regions and health profiles. Standardized reporting (e.g., consistent outcome definitions and data formats) would facilitate future meta-analyses. With ongoing technological advances, future research may also evaluate emerging modalities such as artificial intelligence and AR/VR. Where feasible, future trials could incorporate additional biochemical and physiological markers, extend intervention and follow-up durations, standardize measurement protocols, develop scalable programs across settings, and tailor interventions to specific subgroups.

## 5. Conclusions

The evidence synthesized in this review suggests that digital health technologies, particularly when applied in combined exercise and nutrition programs or in physical activity interventions of moderate to high intensity, may improve MetS-related outcome measures among adolescents. These technologies show potential to improve overall adiposity, central adiposity, and selected cardiometabolic markers through single device, dual device, or multi-component hybrid models, particularly in medium-to-long-term interventions. Combined exercise–nutrition approaches may strengthen the link between behavior change and metabolic health by enabling coordinated targeting of energy intake and expenditure. Although improvements in biochemical outcomes were not consistently observed across studies, the findings underscore the value of digital health technologies for capturing complex interactions among diet, physical activity, and metabolic responses.

From public health and clinical perspectives, these findings support integrating digital health technologies into interventions targeting the prevention and treatment of metabolic syndrome in adolescents. Such approaches may be particularly well suited to youth given high metabolic plasticity and may help address limitations of traditional programs, including limited reach and delayed feedback. They also provide evidence to inform more personalized chronic disease prevention strategies and may facilitate a shift from purely face-to-face delivery toward scalable digital approaches.

Overall, these approaches hold promise for translating digital health technologies into more effective and sustainable prevention and intervention programs for adolescent populations at risk of metabolic disorders. Such efforts may help counter the trend toward earlier onset of metabolic abnormalities and reduce future cardiometabolic disease risk, including cardiovascular disease and type 2 diabetes.

## Figures and Tables

**Table 1:** Detailed Inclusion and Exclusion Criteria.

CATEGORY	INCLUSION CRITERIA
Population	School-aged children and adolescents, including college students (aged 8–22 years)
Study design	Randomized controlled trials (RCTs)
Intervention	Digital health-based interventions targeting diet and/or physical activity, including mobile health applications, wearable activity trackers, telemedicine platforms, social network-based interventions, clinician-oriented digital tools, and VR-assisted exercise programs
Comparator	Usual care, standard lifestyle advice, no digital intervention, wait-list control, non-digital exercise or dietary programs, or alternative non-technological interventions
Outcomes	MetS-related outcomes, including BMI, BMI z-score, WC, WHR, BP, BG, lipid profile, or composite cardiometabolic health measures
Language	English
**CATEGORY**	**EXCLUSION CRITERIA**
Population	Non-adolescent populations aged >22 years or <8 years
Study design	Observational studies, cross-sectional studies, qualitative studies, study protocols, reviews, meta-analyses, editorials, conference abstracts, commentaries, guidelines, letters to the editor, working papers, policy papers and non-randomized or uncontrolled studies
Intervention	Interventions not involving digital health technologies or not related to dietary or physical activity regulation
Outcomes	Studies that did not report any MetS-related outcome measures
Missing data	Studies with incomplete or unclear key metabolic outcome data that could not be obtained from the authors

**Table 2:** Basic characteristics included in the study (specific digital health technology usage, regulatory content, and intervention duration).

Study	Population	Age (Years)	Intervention (Digital Health Technology)	Intervention (Content)	Duration
Lubans et al., 2012 (Australia) [40]	Adolescent girls (low SES)	12–14	Multi-component lifestyle intervention (pedometer + text message support)	Combined intervention ^1^	12 months
Schweitzer et al., 2016 (USA) [41]	College students	18–20	Email-based intervention	Combined intervention ^2^	24 weeks
Chen et al., 2017 (USA) [42]	Overweight/obese adolescents	13–18	Wearable devices + online courses and text message intervention	Combined intervention ^2^	6 months
Bowen-Jallow et al., 2021 (USA) [43]	Obese adolescents (clinic-based)	12–18	Wearable device (Fitbit)	Physical activity only intervention ^3^	18 weeks
Ptomey et al., 2023 (USA) [44]	Adolescents with intellectual disabilities and overweight/obesity	13–21	Remote video sessions + digital self-monitoring (Fitbit and mobile application)	Combined intervention ^4^	18 months
Bicki et al., 2024 (USA) [45]	Youth at cardiovascular risk	8–30	Wearable device (Fitbit) + email or telephone contact	Physical activity only intervention ^3^	6 months
Gómez-Cuesta et al., 2024 (Spain) [46]	Secondary school adolescents	12–16	Step-tracking application	Physical activity only intervention ^5^	10 weeks
Mateo-Orcajada et al., 2024 (Spain) [47]	General adolescent population	12–16	Step-tracking application	Physical activity only intervention ^5^	20 weeks
Sun et al., 2024 (China) [48]	Sedentary adolescents	~18	Wearable metabolic system (K5 Wearable Metabolic System) + heart rate monitor (Polar OH1 Model 2L)	Physical activity only intervention ^6^	8 weeks
Kepper et al., 2024 (USA) [49]	Adolescents with obesity	12–18	Digital counseling tool (PREVENT)	Combined intervention ^2^	3 months
Abd El-Khalek et al., 2025 (Egypt) [50]	Obese adolescent females	12–17	Virtual reality (VR)	Combined intervention ^7^	8 weeks
Ramalho et al., 2025 (Portugal) [51]	Overweight/obese adolescents	13–18	Social network (Facebook) + web-based self-monitoring	Combined intervention ^2^	6 months

Note: ^1^. Nutrition workshops plus physical activity monitoring; ^2^. Dietary guidance plus physical activity monitoring; ^3^. Step-tracking device; ^4^. Enhanced traffic light diet plus physical activity monitoring; ^5^. Step-tracking application; ^6^. High-intensity interval training (HIIT) and moderate-intensity continuous training (MICT); ^7^. Dietary guidance plus aerobic exercise and virtual reality-based games.

**Table 3:** Coverage of metabolic syndrome components across the included studies.

Study	BMI/BMI-z	Central Obesity	BP	BG	BL
Lubans et al., 2012 [40]	✓ ^1,2^	✗	✗	✗	✗
Schweitzer et al., 2016 [41]	✓ ^1^	✓ ^3,5^	✓ ^7^	✗	✗
Chen et al., 2017 [42]	✓ ^1,2^	✓ ^5^	✓ ^7^	✗	✗
Bowen-Jallow et al., 2021 [43]	✓ ^1^	✓ ^3^	✗	✗	✗
Ptomey et al., 2023 [44]	✓ ^1^	✓ ^3^	✗	✗	✗
Bicki et al., 2024 [45]	✗	✗	✓ ^7^	✗	✗
Gómez-Cuesta et al., 2024 [46]	✓ ^1^	✓ ^4^	✗	✗	✗
Mateo-Orcajada et al., 2024 [47]	✓ ^1^	✓ ^3,5^	✗	✗	✗
Sun et al., 2024 [48]	✓ ^1^	✓ ^3,5,6^	✓ ^7^	✓ ^9^	✓ ^10,11,12,13^
Kepper et al., 2024 [49]	✓ ^2^	✗	✓ ^7^	✓ ^8^	✓ ^11^
Abd El-Khalek et al., 2025 [50]	✓ ^1^	✓ ^3,5^	✗	✗	✗
Ramalho et al., 2025 [51]	✓ ^2^	✗	✗	✗	✗

Note: ✓ This measure was not analyzed in the study; ✗ This measure was not analyzed in the study; ^1^. BMI: Body mass index; ^2^. BMI z-score; ^3^. WC: Waist circumference; ^4^. WHtR: Waist-to-height ratio; ^5^. WHR: Waist-to-hip ratio; ^6^. VFA: Visceral fat area; ^7^. BP: Blood pressure; ^8^. BG: Blood glucose; ^9^. HOMA-IR: Homeostatic model assessment of insulin resistance; ^10^. TG: Triglycerides; ^11^. TC: Total cholesterol; ^12^. HDL-C: High-density lipoprotein cholesterol; ^13^. LDL-C: Low-density lipoprotein cholesterol.

**Table 4:** Intervention-related changes in metabolic syndrome components across the included studies.

Study	Intervention Group (*n*)	Control Group (*n*)	Outcome Sample (*n*)	Main Outcomes	Effect Size
Lubans [40]	Combined intervention (*n* = 178)	Usual PE (*n* = 179)	IG (*n* = 141) CG (*n* = 153)	**BMI:** The intervention group showed a downward trend, but neither within-group nor between-group differences were statistically significant. **BMI z-score:** A downward trend was observed in the intervention group, but within-group and between-group differences were not significant.	**BMI:** AMD = −0.19 (95% CI −0.70 to 0.33) **BMI z-score:** AMD = −0.08 (95% CI −0.20 to 0.04)
Schweitze [41]	Combined intervention (*n* = 99)	Usual guidance (*n* = 49)	IG (*n* = 68) CG (*n* = 38)	**BMI:** No significant changes were observed in either group over 24 weeks (*p* = 0.80). **WC and WHR:** No significant between-group differences were observed (*p* = 0.41 and *p* = 0.21). **BP (SBP/DBP):** No significant between-group differences were observed (*p* = 0.92 and *p* = 0.80).	**BMI:** NR **WC and WHR:** NR **BP (SBP/DBP):** NR
Chen [42]	Combined intervention (*n* = 23)	Single physical activity (*n* = 17)	IG (*n* = 21) CG (*n* = 15)	**BMI:** The intervention group showed a significantly greater reduction than the control group (*p* = 0.001). **BMI z-score:** The intervention group showed a significantly greater reduction than the control group (*p* = 0.001). **WHR:** No significant change was observed (*p* > 0.05). **BP:** Overall BP decreased significantly in the intervention group compared with the control group (*p* = 0.001), whereas SBP showed no significant difference.	**BMI:** Cohen’ s d = 0.62 **BMI z-score:** Cohen’s d = 0.34 **WHR:** Cohen’ s d = 0.22 **BP: SBP:** Cohen’ s d = 0.06 **DBP:** Cohen’ s d = 0.21
Bowen- Jallow [43]	Physical activity intervention (*n* = 18)	Usual care (*n* = 30)	IG (*n* = 10) CG (*n* = 23)	**BMI:** No significant between-group difference was observed (*p* = 1.00). **WC:** No significant between-group difference was observed (*p* = 0.83).	**BMI:** SD = 9.39 **WC:** SD = 22.8
Ptomey [44]	Combined intervention (*n* = 36)	Usual diet (*n* = 74)	IG (*n* = 30) CG (*n* = 58)	**BMI:** The intervention group showed a significantly greater reduction than the control group (*p* = 0.03). **WC:** The intervention group showed a significantly greater reduction than the control group (*p* = 0.002).	**BMI:** MD = −1.5 **WC:** MD = −4.7
Bicki [45]	Physical activity intervention (*n* = 42)	Usual care (*n* = 21)	IG (*n* = 26) CG (*n* = 18)	**SBP:** Over 6 months, SBP decreased by −2.3 mmHg in the intervention group (95%CI, −6.5 to 1.8), with no significant between-group difference (*p* = 0.12).	**SBP:** −2.3 mmHg (95% CI −6.5, 1.8)
Gómez- Cuesta [46]	Physical activity intervention (*n* = 280)	Usual PE (*n* = 182)	IG (*n* = 270) CG (*n* = 160)	**BMI:** The intervention group showed no significant change (*p* = 0.344), the control group showed a significant change (*p* = 0.001) **WHtR:** No significant changes were observed in either group (*p* = 0.129 and *p* = 0.187).	**BMI:** η^2^ = 0.002, 0.027 **WHtR:** η^2^ = 0.006, 0.005
Mateo- Orcajada [47]	Physical activity intervention (*n* = 300)	Usual PE (*n* = 165)	IG (*n* = 216) CG (*n* = 141)	**BMI and WC:** No significant between-group differences were observed at any time point (*p* = 0.09, *p* = 0.61). **WHR:** The intervention group showed a significantly greater reduction than the control group (*p* = 0.02).	**BMI:** ηp^2^ = 0.009 **WC:** ηp^2^ = 0.001 **WHR:** ηp^2^ = 0.017
Sun [48]	Physical activity intervention (*n* = 6)	MICT (*n* = 6)	IG (*n* = 6) CG (*n* = 6)	**BMI:** No significant change was observed (*p* > 0.05). **WHR:** A significant reduction was reported in the intervention group (*p* = 0.033). **VFA:** Both the intervention and control groups decreased significantly respectively (*p* = 0.001 and *p* = 0.003). **BP:** SBP and DBP decreased significantly in the intervention group respectively (*p* = 0.018 and *p* = 0.008). **BL:** TG decreased significantly in the intervention group (*p* = 0.004), whereas TC, HDL-C, and LDL-C showed no significant changes (*p* > 0.05). **BG:** No significant between-group difference was observed for BG, and HOMA-IR showed a non-significant downward trend in the intervention group.	**BMI:** NR **WHR:** ES = 0.43 **VFA:** ES = 0.35, 0.49 **BP:** ES = 0.84, 1.76 **TG:** ES = 1.33 **BG:** NR
Kepper [49]	Combined intervention (*n* = 18)	Usual care (*n* = 18)	IG (*n* = 10) CG (*n* = 15)	**BMI z-score:** Both groups showed downward trends, but changes were not statistically significant (95% CI −0.31, 0.20 and 95% CI −0.18, 0.10). **BP:** SBP decreased significantly in the intervention group (within-group *p* = 0.009; between-group *p* = 0.001). DBP decreased significantly within the intervention group (within-group *p* = 0.009), but the between-group difference was not significant. **BG and TC:** Downward trends were reported; however, due to high missingness, statistical significance testing was not performed.(95% CI −36.83, 20.08 and 95% CI −21.46, 21.29, 95% CI −366.45, 389.27 and 95% CI −46.92, 58.32)	**BMI z-score:** IG: (95% CI −0.31, 0.20), CG: (95% CI −0.18, 0.10) **SBP:** IG: (95% CI −18.46, −2.98), CG: (95% CI −5.06, 15.17) **DBP:** IG: (95% CI −12.86, −2.19) CG: (95% CI −12.07, 2.68) **BG:** IG: (95% CI −36.83, 20.08), CG: (95% CI −21.46, 21.29) **TC:** IG: (95% CI −366.45, 389.27), CG: (95% CI −46.92, 58.32)
Abd El- Khalek [50]	Combined intervention (*n* = 50)	No VR equipment (*n* = 50)	IG (*n* = 50) CG (*n* = 50)	**BMI:** Both groups decreased significantly post-intervention (within-group *p* < 0.001), and the reduction was significantly greater in the intervention group (between-group *p* = 0.001). **WC and WHR:** Both groups improved significantly (within-group *p* < 0.001), but between-group differences were not significant.	**BMI:** Cohen’ s d = 1.73 **WC:** Cohen’ s d = 0.23 **WHR:** Cohen’ s d = 0.32
Ramalho [51]	Combined intervention (*n* = 69)	Usual guidance (*n* = 66)	IG (*n* = 38) CG (*n* = 39)	**BMI z-score:** Both groups decreased significantly over time (*p* = 0.006), with no significant group-by-time interaction.	**BMI z-score:** ηp^2^ = 0.116

**Table 5:** Intervention designs across studies sorted by intervention timing.

Study	Duration	Intervention (Content)	Main Outcomes
Sun et al., [48]	8 weeks	Physical activity only intervention (HIIT)	**BMI** ^1,5,6^ **WHR** ^3^ **VFA** ^3,4^ **BP** ^3^ **BL:** TG ^3^, (TC, HDL-C, and LDL-C) ^1,3,4^ **BG:** BG ^1,3,4^, HOMA-IR ^7^
Abd El-Khalek et al., [50]	8 weeks	Combined intervention (Dietary guidance plus aerobic exercise and virtual reality-based games)	**BMI** ^2,3,4^ **WC and WHR** ^1,3,4^
Gómez-Cuesta et al., [46]	10 weeks	Physical activity only intervention (Step-tracking application)	**BMI** ^3^ **WHtR** ^1,3,4^
Kepper et al., [49]	3 months	Combined intervention (Dietary guidance plus physical activity monitoring)	**BMI z-score** ^8^ **BP:** SBP ^2,3^, DBP ^3^ **TC** ^8^ **BG** ^8^
Bowen-Jallow et al., [43]	18 weeks	Physical activity only intervention (Step-tracking device)	**BMI** ^1^ **WC** ^1^
Mateo-Orcajada et al., [47]	20 weeks	Physical activity only intervention (Step-tracking application)	**BMI** ^1^ **WC** ^1^ **WHR**^2^
Schweitzer et al., [41]	24 weeks	Combined intervention (Dietary guidance plus physical activity monitoring)	**BMI** ^5,6^ **WC and WHR** ^5,6^ **BP** (SBP/DBP) ^5,6^
Ramalho et al., [51]	6 months	Combined intervention (Dietary guidance plus physical activity monitoring)	**BMI z-score** ^3,4^
Chen et al., [42]	6 months	Combined Intervention (Dietary guidance plus physical activity monitoring)	**BMI** ^2^ **BMI z-score** ^2^ **WHR** ^5,6^ **BP:** Overall BP ^2^, SBP ^1^
Bicki et al., 2024 (USA) [45]	6 months	Physical activity only intervention (Step-tracking device)	**SBP** ^1,7^
Lubans et al., [40]	12 months	Combined intervention (Nutrition workshops plus physical activity monitoring)	**BMI** ^1,6,7^ **BMI z-score** ^1,6,7^
Ptomey et al., [44]	18 months	Combined Intervention (Enhanced traffic light diet plus physical activity monitoring)	**BMI** ^2^ **WC** ^2^

Note: ^1^. No significant difference between groups; ^2^. Significant differences between groups; ^3^. Significant changes within the intervention group; ^4^. Significant changes within the control group; ^5^. No significant changes occurred within the intervention group; ^6^. No significant changes occurred within the intervention group; ^7^. Intervention group showed a downward trend but this was not statistically significant; ^8^. Two groups showed a downward trend but this was not statistically significant.

## Data Availability

No new data were created or analyzed in this study. Data sharing is not applicable to this article.

## Supplementary Materials

The following supporting information can be downloaded at: https://www.mdpi.com/article/10.3390/metabo16020106/s1, File S1: Search strategy; Table S1: Detailed characteristics of the 12 studies analyze; Table S2: Concise version Characteristics and outcomes included in the study; Table S3: Final Risk Assessment of 12 Studies; Table S4: PRISMA_2020_checklist.

## Abbreviations

The following abbreviations are used in this manuscript:

MetS	Metabolic Syndrome
BMI	Body Mass Index
BP	Blood Pressure
SBP	Systolic Blood Pressure
DBP	Diastolic Blood Pressure
BG	Blood Glucose
BL	Blood Lipids
WC	Waist Circumference
WHtR	Waist-to-height Ratio
WHR	Waist-to-hip Ratio
VFA	Visceral Fat Area
HOMA-IR	Homeostatic Model Assessment of Insulin Resistance
TG	Triglycerides
TC	Total Cholesterol
HDL-C	High-density Lipoprotein Cholesterol
LDL-C	Low-density Lipoprotein Cholesterol
VR	Virtual Reality
AR	Augmented Reality
HIIT	High-intensity interval training
MICT	Moderate-intensity continuous training

## References

[1] Xie Z.M., Yu C.L., Cui Q.M., Zhao X.R., Zhuang J.C., Chen S.Q., Guan H.X., Li J. (2025). Global Burden of the Key Components of Cardiovascular-Kidney-Metabolic Syndrome. J. Am. Soc. Nephrol..

[2] Accili D., Deng Z.B., Liu Q.L. (2025). Insulin resistance in type 2 diabetes mellitus. Nat. Rev. Endocrinol..

[3] Valsamakis G., Chetty R., Anwar A., Banerjee A.K., Barnett A., Kumar S. (2004). Association of simple anthropometric measures of obesity with visceral fat and the metabolic syndrome in male Caucasian and Indo-Asian subjects. Diabet. Med..

[4] Huang Q.W., Yang D.Z., Deng H.R., Liang H., Zheng X.Y., Yan J.H., Xu W., Liu X.W., Yao B., Luo S.H. (2022). Association between Metabolic Syndrome and Microvascular Complications in Chinese Adults with Type 1 Diabetes Mellitus. Diabetes Metab. J..

[5] Ford E.S. (2005). Prevalence of the metabolic syndrome defined by the International Diabetes Federation among adults in the U.S. Diabetes Care.

[6] Ko S.H., Kim H.S. (2020). Menopause-Associated Lipid Metabolic Disorders and Foods Beneficial for Postmenopausal Women. Nutrients.

[7] Scaglione S., Di Chiara T., Daidone M., Tuttolomondo A. (2025). Effects of the Mediterranean Diet on the Components of Metabolic Syndrome Concerning the Cardiometabolic Risk. Nutrients.

[8] Gui J.F., Li Y.Q., Liu H.Y., Guo L.L., Li J.L., Lei Y.X., Li X.P., Sun L., Yang L., Yuan T. (2023). Obesity- and lipid-related indices as a predictor of obesity metabolic syndrome in a national cohort study. Front. Public Health.

[9] Fahed G., Aoun L., Zerdan M.B., Allam S., Zerdan M.B., Bouferraa Y., Assi H.I. (2022). Metabolic Syndrome: Updates on Pathophysiology and Management in 2021. Int. J. Mol. Sci..

[10] da Silva A.A., do Carmo J.M., Li X., Wang Z., Mouton A.J., Hall J.E. (2020). Role of Hyperinsulinemia and Insulin Resistance in Hypertension: Metabolic Syndrome Revisited. Can. J. Cardiol..

[11] Li W.P., Shen C.N., Kong W.Y., Zhou X.H., Fan H.M., Zhang Y.Z., Liu Z.M., Zheng L. (2024). Association between the triglyceride glucose-body mass index and future cardiovascular disease risk in a population with Cardiovascular-Kidney-Metabolic syndrome stage 0-3: A nationwide prospective cohort study. Cardiovasc. Diabetol..

[12] Bonadonna R.C., Cucinotta D., Fedele D., Riccardi G., Tiengo A., Metascreen Writing C. (2006). The metabolic syndrome is a risk indicator of microvascular and macrovascular complications in diabetes—Results from Metascreen, a multicenter diabetes clinic-based survey. Diabetes Care.

[13] Martemucci G., Fracchiolla G., Muraglia M., Tardugno R., Dibenedetto R.S., D’Alessandro A.G. (2023). Metabolic Syndrome: A Narrative Review from the Oxidative Stress to the Management of Related Diseases. Antioxidants.

[14] Wisse B.E. (2004). The inflammatory syndrome: The role of adipose tissue cytokines in metabolic disorders linked to obesity. J. Am. Soc. Nephrol..

[15] Martínez-Vizcaino V., Ortega F.B., Solera-Martínez M., Ruiz J.R., Labayen I., Eensoo D., Harro J., Loit H.M., Veidebaum T., Sjöström M. (2011). Stability of the factorial structure of metabolic syndrome from childhood to adolescence: A 6-year follow-up study. Cardiovasc. Diabetol..

[16] Wentzel A., Mabhida S.E., Ndlovu M., Mokoena H., Esterhuizen B., Sekgala M.D., Dludla P.V., Kengne A.P., McHiza Z.J. (2025). Prevalence of metabolic syndrome in children and adolescents with obesity: A systematic review and meta-analysis. Obesity.

[17] Anderer S. (2025). Severe Pediatric Obesity Is Growing, Tied to Higher Metabolic Risks. JAMA.

[18] Guthold R., Stevens G.A., Riley L.M., Bull F.C. (2020). Global trends in insufficient physical activity among adolescents: A pooled analysis of 298 population-based surveys with 1.6 million participants. Lancet Child. Adolesc. Health.

[19] Chaput J.P., Willumsen J., Bull F., Chou R., Ekelund U., Firth J., Jago R., Ortega F.B., Katzmarzyk P.T. (2020). 2020 WHO guidelines on physical activity and sedentary behaviour for children and adolescents aged 5-17years: Summary of the evidence. Int. J. Behav. Nutr. Phys. Act..

[20] Ruiz L.D., Zuelch M.L., Dimitratos S.M., Scherr R.E. (2020). Adolescent Obesity: Diet Quality, Psychosocial Health, and Cardiometabolic Risk Factors. Nutrients.

[21] Roden M., Shulman G.I. (2019). The integrative biology of type 2 diabetes. Nature.

[22] Hajhosseiny R., Matthews G.K., Lip G.Y.H. (2015). Metabolic syndrome, atrial fibrillation, and stroke: Tackling an emerging epidemic. Heart Rhythm.

[23] Braffett B.H., Bebu I., Lorenzi G.M., Martin C.L., Perkins B.A., Gubitosi-Klug R., Nathan D.M., Grp D.E.R. (2025). The NIDDK Takes on the Complications of Type 1 Diabetes: The Diabetes Control and Complications Trial/Epidemiology of Diabetes Interventions and Complications (DCCT/EDIC) Study. Diabetes Care.

[24] Perng W., Oken E., Dabelea D. (2019). Developmental overnutrition and obesity and type 2 diabetes in offspring. Diabetologia.

[25] Sostaric A., Jenko B., Kozjek N.R., Ovijac D., Suput D., Milisav I., Dolzan V. (2019). Detection of metabolic syndrome burden in healthy young adults may enable timely introduction of disease prevention. Arch. Med. Sci..

[26] Franco J.V.A., Guo Y., Bongaerts B., Metzendorf M.I., Hindemit J., Aqra Z., Alhalahla M., Tapinova K., Villegas Arbelaez E., Alade O.T. (2025). Multimodal health behaviour-changing interventions for adolescents living with obesity. Cochrane Database Syst. Rev..

[27] Peiris C.L., van Namen M., O’Donoghue G. (2021). Education-based, lifestyle intervention programs with unsupervised exercise improve outcomes in adults with metabolic syndrome. A systematic review and meta-analysis. Rev. Endocr. Metab. Disord..

[28] Fernando K., Connolly D., Darcy E., Evans M., Hinchliffe W., Holmes P., Strain W.D. (2025). Advancing Cardiovascular, Kidney, and Metabolic Medicine: A Narrative Review of Insights and Innovations for the Future. Diabetes Ther..

[29] Huh U., Tak Y.J., Song S., Chung S.W., Sung S.M., Lee C.W., Bae M., Ahn H.Y. (2019). Feedback on Physical Activity Through a Wearable Device Connected to a Mobile Phone App in Patients with Metabolic Syndrome: Pilot Study. JMIR mHealth uHealth.

[30] Sakane N., Suganuma A., Domichi M., Sukino S., Abe K., Fujisaki A., Kanazawa A., Sugimoto M. (2023). The Effect of a mHealth App (KENPO-app) for Specific Health Guidance on Weight Changes in Adults with Obesity and Hypertension: Pilot Randomized Controlled Trial. JMIR mHealth uHealth.

[31] Szálka B., Vassányi I., Köteles E.M., Szabó L.A., Lada S., Bolgár T., Korom A., Abrahám J., Bilicki V., Barnai M. (2024). Factors of Weight Loss for Telemedically Supported Metabolic Syndrome Patients in a Controlled Trial. Appl. Sci..

[32] Modi K., Singh I., Kumar Y. (2023). A Comprehensive Analysis of Artificial Intelligence Techniques for the Prediction and Prognosis of Lifestyle Diseases. Arch. Comput. Method Eng..

[33] Huynh P., Fleisch E., Braendle M., Kowatsch T., Jovanova M. (2024). Digital health technologies for metabolic disorders in older adults: A scoping review protocol. BMJ Open.

[34] Zhang Y., Ngai F.W., Yang Q.L., Xie Y.J. (2025). Effectiveness of Digital Health Interventions on Sedentary Behavior Among Patients with Chronic Diseases: Systematic Review and Meta-Analysis. JMIR mHealth uHealth.

[35] Delva S., Mendez K.J.W., Cajita M., Koirala B., Shan R.Z., Wongvibulsin S., Vilarino V., Gilmore D.R., Han H.R. (2021). Efficacy of Mobile Health for Self-management of Cardiometabolic Risk Factors A Theory-Guided Systematic Review. J. Cardiovasc. Nurs..

[36] Oliveira A.C.N., Guariente S.M.M., Zazula R., Mesas A.E., Oliveira C.E.C., Reiche E.M.V., Nunes S.O.V. (2022). Hybrid and Remote Psychosocial Interventions Focused on Weight and Sedentary Behavior Management Among Patients with Severe Mental Illnesses: A Systematic Review. Psychiatr. Q..

[37] Sumengen A.A., Savas E.H., Ay A., Koyuncu I.E., Erkul M., Semerci R. (2024). Impact of Technology on Physical Activity Levels of Childhood Cancer Survivors: A Systematic Review. Semin. Oncol. Nurs..

[38] Page M.J., McKenzie J.E., Bossuyt P.M., Boutron I., Hoffmann T.C., Mulrow C.D., Shamseer L., Tetzlaff J.M., Akl E.A., Brennan S.E. (2021). The PRISMA 2020 statement: An updated guideline for reporting systematic reviews. BMJ.

[39] Folgori L., Tersigni C., Hsia Y., Kortsalioudaki C., Heath P., Sharland M., Bielicki J. (2018). The relationship between Gram-negative colonization and bloodstream infections in neonates: A systematic review and meta-analysis. Clin. Microbiol. Infect..

[40] Lubans D.R., Morgan P.J., Okely A.D., Dewar D., Collins C.E., Batterham M., Callister R., Plotnikoff R.C. (2012). Preventing Obesity Among Adolescent Girls. Arch. Pediatr. Adolesc. Med..

[41] Schweitzer A.L., Ross J.T., Klein C.J., Lei K.Y., Mackey E.R. (2016). An Electronic Wellness Program to Improve Diet and Exercise in College Students: A Pilot Study. JMIR Res. Protoc..

[42] Chen J.-L., Guedes C.M., Cooper B.A., Lung A.E. (2017). Short-Term Efficacy of an Innovative Mobile Phone Technology-Based Intervention for Weight Management for Overweight and Obese Adolescents: Pilot Study. Interact. J. Med. Res..

[43] Bowen-Jallow K., Nunez-Lopez O., Wright A., Fuchs E., Ahn M., Lyons E., Jupiter D., Berry L., Suman O., Radhakrishnan R.S. (2021). Wearable Activity Tracking Device Use in an Adolescent Weight Management Clinic: A Randomized Controlled Pilot Trial. J. Obes..

[44] Ptomey L.T., Washburn R.A., Goetz J.R., Sullivan D.K., Gibson C.A., Mayo M.S., Krebill R., Gorczyca A.M., Honas J.J., Rice A.M. (2023). A randomized trial comparing diet and delivery strategies for weight management in adolescents with intellectual disabilities. Pediatr. Obes..

[45] Bicki A.C., Seth D., McCulloch C.E., Lin F., Ku E. (2024). Use of activity trackers to improve blood pressure in young people at risk for cardiovascular disease: A pilot randomized controlled trial. Pediatr. Nephrol..

[46] Gomez-Cuesta N., Mateo-Orcajada A., Merono L., Abenza-Cano L., Vaquero-Cristobal R. (2024). A mobile app-based intervention improves anthropometry, body composition and fitness, regardless of previous active-inactive status: A randomized controlled trial. Front. Public Health.

[47] Mateo-Orcajada A., Vaquero-Cristoba R., Mota J., Abenza-Cano L. (2024). Physical Activity, Body Composition, and Fitness Variables inAdolescents After Periods of Mandatory, Promoted orNonmandatory, Nonpromoted Use of Step Tracker Mobile Apps:Randomized Controlled Trial. JMIR mHealth uHealth.

[48] Sun F., Williams C.A., Sun Q., Hu F., Zhang T. (2024). Effect of eight-week high-intensity interval training versus moderate-intensity continuous training programme on body composition, cardiometabolic risk factors in sedentary adolescents. Front. Physiol..

[49] Kepper M., Walsh-Bailey C., Miller Z.M., Zhao M., Zucker K., Gacad A., Herrick C., White N.H., Brownson R.C., Foraker R.E. (2024). The Impact of Behavior Change Counseling Delivered via a Digital Health Tool Versus Routine Care Among Adolescents with Obesity: Pilot Randomized Feasibility Study. JMIR Form. Res..

[50] Abd El-khalek W.O.A., Abdulrahman R.S., Mahmoud H., Saeed D., Elias M.A.G., Ebid A.A., El-Fiky A., Alayat M.S., Abdulrahman Y.A.F., Hassan M.G. (2025). Effects of dietary advice, aerobic exercise, and virtual reality games on the quality of life of obese adolescent females. Sport TK.

[51] Ramalho S.M., Saint-Maurice P.F., Silva D., Mansilha H.F., Conceicao E. (2025). Feasibility and Effectiveness of a Social Network-Based Intervention for Adolescents Undergoing Weight Loss Treatment: A Randomized Controlled Trial. Nutrients.

[52] Kim M., Kim J. (2022). Cardiometabolic risk factors and metabolic syndrome based on severity of obesity in Korean children and adolescents: Data from the Korea National Health and Nutrition Examination Survey 2007–2018. Ann. Pediatr. Endocrinol. Metab..

[53] Sisay B.G., Jima B.R., Habtamu M., Gebru N.W., Hassen H.Y. (2023). Predictive ability of anthropometric indices in identifying metabolic syndrome among US adolescents 10 to 19 years old: Analysis from the National Health and Nutrition Examination Survey 2011 to 2018 data set. Nutrition.

[54] Javidi H., Mariam A., Alkhaled L., Pantalone K.M., Rotroff D.M. (2024). An interpretable predictive deep learning platform for pediatric metabolic diseases. J. Am. Med. Inf. Assoc..

[55] Jayedi A., Soltani S., Zargar M.S., Khan T.A., Shab-Bidar S. (2020). Central fatness and risk of all cause mortality: Systematic review and dose-response meta-analysis of 72 prospective cohort studies. BMJ.

[56] Guzmán-García J.M., Romero-Saldaña M., Molina-Recio G., Fonseca-del Pozo F.J., Raya-Cano E., Molina-Luque R. (2023). Diagnostic accuracy of anthropometric indices for metabolically healthy obesity in child and adolescent population. Pediatr. Res..

[57] González-Domínguez A., Domínguez-Riscart J., Savolainen O., Lechuga-Sancho A., Landberg R., González-Domínguez R. (2024). Identifying metabotypes of insulin resistance severity in children with metabolic syndrome. Cardiovasc. Diabetol..

[58] Song K., Lee E., Lee H.S., Lee H., Lee J.W., Chae H.W., Kwon Y.J. (2025). Comparison of SPISE and METS-IR and Other Markers to Predict Insulin Resistance and Elevated Liver Transaminases in Children and Adolescents. Diabetes Metab. J..

[59] Sharif H., Sheikh S.S., Seemi T., Naeem H., Khan U., Jan S.S. (2024). Metabolic syndrome and obesity among marginalised school-going adolescents in Karachi, Pakistan: A cross-sectional study. Lancet Reg. Health Southeast Asia.

[60] Noce A., Di Lauro M., Di Daniele F., Zaitseva A.P., Marrone G., Borboni P., Di Daniele N. (2021). Natural Bioactive Compounds Useful in Clinical Management of Metabolic Syndrome. Nutrients.

[61] Nayak S.S., Kuriyakose D., Polisetty L.D., Patil A.A., Ameen D., Bonu R., Shetty S.P., Biswas P., Ulrich M.T., Letafatkar N. (2024). Diagnostic and prognostic value of triglyceride glucose index: A comprehensive evaluation of meta-analysis. Cardiovasc. Diabetol..

[62] Lister N.B., Baur L.A., House E.T., Alexander S., Brown J., Collins C.E., Cowell C.T., Day K., Garnett S.P., Gow M.L. (2024). Intermittent Energy Restriction for Adolescents with Obesity:The Fast Track to Health Randomized Clinical Trial. JAMA Pediatr..

[63] Graversen P., Abildstrom S.Z., Jespersen L., Borglykke A., Prescott E. (2016). Cardiovascular risk prediction: Can Systematic Coronary Risk Evaluation (SCORE) be improved by adding simple risk markers? Results from the Copenhagen City Heart Study. Eur. J. Prev. Cardiol..

[64] Moldovan A., Waldman Y.Y., Brandes N., Linial M. (2021). Body Mass Index and Birth Weight Improve Polygenic Risk Score for Type 2 Diabetes. J. Pers. Med..

[65] Gao C.N., Meng X., Liu W., Qi Q.J., Yan Y.K. (2024). Identification of sensitive periods of weight status transition over the lifespan in Chinese population. BMC Med..

[66] Kim H.L., Joh H.S., Lim W.H., Seo J.B., Kim S.H., Zo J.H., Kim M.A. (2023). Associations of Estimated Pulse Wave Velocity with Body Mass Index and Waist Circumference among General Korean Adults. Metabolites.

[67] Qiao T.T., Luo T., Pei H.L., Yimingniyazi B., Aili D., Aimudula A., Zhao H., Zhang H.W., Dai J.H., Wang D.L. (2022). Association between abdominal obesity indices and risk of cardiovascular events in Chinese populations with type 2 diabetes: A prospective cohort study. Cardiovasc. Diabetol..

[68] Martinez-Dominguez P., Gomez-Aviles P., Bautista-García K., Antonio-Villa N.E., Guerra E.C., Almeda-Valdes P., Martagón A.J., Munoz A.C., Santa-Ana-Bayona M.J., Alexanderson E. (2025). Visceral adipose tissue mediates the relationship between left ventricular global longitudinal strain and insulin resistance among adults living with type 2 diabetes. Cardiovasc. Diabetol..

[69] Kolb H. (2022). Obese visceral fat tissue inflammation: From protective to detrimental?. BMC Med..

[70] Ko S.H., Jung Y. (2021). Energy Metabolism Changes and Dysregulated Lipid Metabolism in Postmenopausal Women. Nutrients.

[71] Szablewski L. (2024). Insulin Resistance: The Increased Risk of Cancers. Curr. Oncol..

[72] Agarwal S.K., Misra A., Aggarwal P., Bardia A., Goel R., Vikram N.K., Wasir J.S., Hussain N., Ramachandran K., Pandey R.M. (2009). Waist Circumference Measurement by Site, Posture, Respiratory Phase, and Meal Time: Implications for Methodology. Obesity.

[73] Wang C.Y., Liu M.H., Chen Y.C. (2010). Intrarater reliability and the value of real change for waist and hip circumference measures by a novice rater. Percept. Mot. Skills.

[74] Liao Z.J., Wang J., Chen Y.R., Li W.Q., Xie X.H., Zhang T., Liu G.S., Chen F.F. (2025). Associations of Body Mass Index Growth Rates and Body Composition with Cardiometabolic Risks in Chinese Preschool Children. J. Clin. Endocrinol. Metab..

[75] Chen L., Liu Q., Xu F.L., Wang F.M., Luo S.Q., An X.Z., Chen J.Y., Tang N., Jiang X.P., Liang X.H. (2024). Effect of physical activity on anxiety, depression and obesity index in children and adolescents with obesity: A meta-analysis. J. Affect. Disord..

[76] Rajjo T., Mohammed K., Alsawas M., Ahmed A.T., Farah W., Asi N., Almasri J., Prokop L.J., Murad M.H. (2017). Treatment of Pediatric Obesity: An Umbrella Systematic Review. J. Clin. Endocrinol. Metab..

[77] Solomon T.P.J. (2018). Sources of Inter-individual Variability in the Therapeutic Response of Blood Glucose Control to Exercise in Type 2 Diabetes: Going Beyond Exercise Dose. Front. Physiol..

[78] Nusca A., Tuccinardi D., Albano M., Cavallaro C., Ricottini E., Manfrini S., Pozzilli P., Di Sciascio G. (2018). Glycemic variability in the development of cardiovascular complications in diabetes. Diabetes Metab. Res. Rev..

[79] He L.X., Liu M.H., Zhuang X.D., Guo Y., Wang P., Zhou Z.M., Chen Z.H., Peng L.Y., Liao X.X. (2024). Effect of Intensive Lifestyle Intervention on Cardiovascular Risk Factors: Analysis from the Perspective of Long-Term Variability. J. Am. Heart Assoc..

[80] Headland M., Clifton P.M., Carter S., Keogh J.B. (2016). Weight-Loss Outcomes: A Systematic Review and Meta-Analysis of Intermittent Energy Restriction Trials Lasting a Minimum of 6 Months. Nutrients.

[81] Feng J.W., Zhang Q.H., Chen B.Y., Chen J.P., Wang W.J., Hu Y.H., Yu J.B., Huang H.M. (2024). Effects of high-intensity intermittent exercise on glucose and lipid metabolism in type 2 diabetes patients: A systematic review and meta-analysis. Front. Endocrinol..

[82] Takano K., Oba T., Katahira K., Kimura K. (2024). Deconstructing Fitbit to Specify the Effective Features in Promoting Physical Activity Among Inactive Adults: Pilot Randomized Controlled Trial. JMIR mHealth uHealth.

[83] Verhulst I., Woods A., Whittaker L., Bennett J., Dalton P. (2021). Do VR and AR versions of an immersive cultural experience engender different user experiences?. Comput. Hum. Behav..

[84] Leme A.C.B., Haines J., Tang L.S., Dunker K.L.L., Philippi S.T., Fisberg M., Ferrari G.L., Fisberg R.M. (2020). Impact of Strategies for Preventing Obesity and Risk Factors for Eating Disorders among Adolescents: A Systematic Review. Nutrients.

[85] Hodder R.K., O’Brien K.M., Al-Gobari M., Flatz A., Borchard A., Klerings I., Clinton-McHarg T., Kingsland M., von Elm E. (2025). Interventions implemented through sporting organisations for promoting healthy behaviour or improving health outcomes (Review). Cochrane Database Syst Rev..

[86] Li S.J., Zhou Y., Tang Y., Ma H.M., Zhang Y.Y., Wang A.Q., Tang X.Y., Pei R.Y., Piao M.H. (2025). Behavior Change Resources Used in Mobile App-Based Interventions Addressing Weight, Behavioral, and Metabolic Outcomes in Adults with Overweight and Obesity: Systematic Review and Meta-Analysis of Randomized Controlled Trials. JMIR mHealth uHealth.

[87] Leziak A., Lipina J., Reclik M., Kocelak P. (2025). Dietary Modulation of Metabolic Health: From Bioactive Compounds to Personalized Nutrition. Metabolites.

[88] Hall K.D., Guo J. (2017). Obesity Energetics: Body Weight Regulation and the Effects of Diet Composition. Gastroenterology.

[89] Martins C., Roekenes J., Salamati S., Gower B.A., Hunter G.R. (2020). Metabolic adaptation is an illusion, only present when participants are in negative energy balance. Am. J. Clin. Nutr..

[90] Terranova C.O., Brakenridge C.L., Lawler S.P., Eakin E.G., Reeves M.M. (2015). Effectiveness of lifestyle-based weight loss interventions for adults with type 2 diabetes: A systematic review and meta-analysis. Diabetes Obes. Metab..

[91] Moschonis G., Siopis G., Jung J., Eweka E., Willems R., Kwasnicka D., Asare B.Y.A., Kodithuwakku V., Verhaeghe N., Vedanthan R. (2023). Effectiveness, reach, uptake, and feasibility of digital health interventions for adults with type 2 diabetes: A systematic review and meta-analysis of randomised controlled trials. Lancet Digit. Health.

