# Supplementary material for: How Emerging Digital Health Technologies Based on Dietary and Physical Activity Regulation Improve Metabolic Syndrome-Related Outcomes in Adolescents: A Systematic Review
Source: Metabolites. 2026 Feb 2;16(2):106. doi: 10.3390/metabo16020106 (PMC12942388; doi:10.3390/metabo16020106)
Supplement: Supplementary file 1 [file metabolites-16-00106-s001.zip › Supplementary File S1 search strategy.pdf]

Dietary intervention

#1 TS=(Diet Therapy *OR* Diet Therapies *OR* Dietary Modification *OR* Dietary Modifications *OR* Diet Modification *OR* Diet Modifications *OR* Restriction Diet Therapies *OR* Restrictive Diet Therapies *OR* Restriction Diet Therapy *OR* Restrictive Diet Therapy *OR* Dietary Restriction *OR* Dietary Restrictions *OR* Diets)

Emerging digital health technologies

#2 TS=(Self-Help Devices *OR* Self-Help Device *OR* Self Help Devices *OR* Assistive Devices *OR* Assistive Device *OR* Assistive Technology *OR* Assistive Technologies *OR* Biomedical Enhancement *OR* Enhancement Technologies *OR* Telemedicine *OR* Virtual Medicine *OR* Mobile Health OR mHealth OR Telehealth OR eHealth OR Digital Health OR Digital Health Technology *OR* Digital Health Technologies *OR* Mobile Applications *OR* Mobile Application *OR* Mobile App *OR* Portable Electronic App *OR* Portable Electronic Applications *OR* Portable Electronic Application *OR* Portable Software Apps *OR* Portable Software Applications *OR* Smartphone Apps *OR* Smartphone App *OR* Wearable Electronic Device *OR* Wearable Devices *OR* Wearable Device *OR* Wearable Technology *OR* Wearable Technologies *OR* Electronic Skin *OR* Wearable Computer *OR* Wearable Computers)

Sports

#3 TS=(Exercise *OR* Sports *OR* Motor Activity *OR* Physical Exertion *OR* Physical Effort *OR* Exercises *OR* Physical Exercise *OR* Physical Exercises *OR* Aerobic Exercise *OR* Aerobic Exercises *OR* Isometric Exercises *OR* Isometric Exercise *OR* Acute Exercise *OR* Acute Exercises *OR* Exercise Training *OR* Exercise Trainings *OR* Physical Activity *OR* Physical Activities)

Metabolic syndrome

#4 TS=(Metabolic Syndrome *OR* Blood Pressure *OR* Triglycerides *OR* Cholesterol, HDL *OR* Blood Glucose *OR* Insulin *OR* Body Weight *OR* Waist-Hip Ratio *OR* Waist Circumference *OR* Body Weights *OR* Blood Sugar *OR* High Density Lipoprotein Cholesterol *OR* Metabolic Syndromes *OR* Reaven Syndrome X *OR* Metabolic Syndrome X *OR* Insulin Resistance Syndrome X *OR* Metabolic Cardiovascular Syndrome *OR* Metabolic X Syndrome *OR* Dysmetabolic Syndrome X *OR* Cardiometabolic Syndrome *OR* Cardiometabolic Syndromes)

Young people

#5 TS=(Adolescent *OR* Adolescence *OR* Female Adolescent *OR* Male Adolescent *OR* Youth *OR* Youths *OR* Teens *OR* Teen *OR* Teenagers *OR* Teenager *OR* Young Adult *OR* Young Adults *OR* college student *OR* undergraduate)

WoS

((TS=(Diet Therapy OR Diet Therapies OR Dietary Modification OR Dietary Modifications OR Diet Modification OR Diet Modifications OR Restriction Diet Therapies OR Restrictive Diet Therapies OR Restriction Diet Therapy OR Restrictive Diet Therapy OR Dietary Restriction OR Dietary Restrictions OR Diets) AND TS=(Exercise OR Sports OR Motor Activity OR Physical Exertion OR Physical Effort OR Exercises OR Physical Exercise OR Physical Exercises OR Aerobic Exercise OR Aerobic Exercises OR Isometric Exercises OR Isometric Exercise OR Acute Exercise OR Acute Exercises OR Exercise Training OR Exercise Trainings OR Physical Activity OR Physical Activities)) AND TS=(Self-Help Devices OR Self-Help Device OR Self Help Devices OR Assistive Devices OR Assistive Device OR Assistive Technology OR Assistive Technologies OR Biomedical Enhancement OR Enhancement Technologies OR Telemedicine OR Virtual Medicine OR Mobile Health OR mHealth OR Telehealth OR eHealth OR Digital Health OR Digital Health Technology OR Digital Health Technologies OR Mobile Applications OR Mobile Application OR Mobile App OR Portable Electronic App OR Portable Electronic Applications OR Portable Electronic Application OR Portable Software Apps OR Portable Software Applications OR Smartphone Apps OR Smartphone App OR Wearable Electronic Device OR Wearable Devices OR Wearable Device OR Wearable Technology OR Wearable Technologies OR Electronic Skin OR Wearable Computer OR Wearable Computers) AND TS=(Metabolic Syndrome OR Blood Pressure OR Triglycerides OR Cholesterol, HDL OR Blood Glucose OR Insulin OR Body Weight OR Waist-Hip Ratio OR Waist Circumference OR Body Weights OR Blood Sugar OR High Density Lipoprotein Cholesterol OR Metabolic Syndromes OR Reaven Syndrome X OR Metabolic Syndrome X OR Insulin Resistance Syndrome X OR Metabolic Cardiovascular Syndrome OR Metabolic X Syndrome OR Dysmetabolic Syndrome X OR Cardiometabolic Syndrome OR Cardiometabolic Syndromes)) AND (TS=(Adolescent *OR* Adolescence *OR* Female Adolescent *OR* Male Adolescent *OR* Youth *OR* Youths *OR* Teens *OR* Teen *OR* Teenagers *OR* Teenager *OR* Young Adult *OR* Young Adults *OR* college student *OR* undergraduate))

PubMed

(((#1) AND(#4)) OR ((#2) AND (#3))) AND (#5)

(((Diet Therapy[MeSH] OR Diets[MeSH] OR Diet Therapies[Title/Abstract] OR Dietary Modification[Title/Abstract] OR Dietary Modifications[Title/Abstract] OR Diet Modification[Title/Abstract] OR Diet Modifications[Title/Abstract] OR Restriction Diet Therapies[Title/Abstract] OR Restrictive Diet Therapies[Title/Abstract] OR Restriction Diet Therapy[Title/Abstract] OR Restrictive Diet Therapy[Title/Abstract] OR Dietary Restriction[Title/Abstract] OR Dietary Restrictions[Title/Abstract]) AND (Exercise[MeSH] OR Sports[MeSH] OR Motor Activity[MeSH] OR Physical Exertion[MeSH] OR Physical Effort[Title/Abstract] OR Exercises[Title/Abstract] OR Physical Exercise[Title/Abstract] OR Physical Exercises[Title/Abstract] OR Aerobic Exercise[Title/Abstract] OR Aerobic Exercises[Title/Abstract] OR Isometric Exercises[Title/Abstract] OR Isometric Exercise[Title/Abstract] OR Acute Exercise[Title/Abstract] OR Acute Exercises[Title/Abstract] OR Exercise Training[Title/Abstract] OR Exercise Trainings[Title/Abstract] OR Physical Activity[Title/Abstract] OR Physical Activities[Title/Abstract])) AND (Self-Help Devices[MeSH] OR Biomedical Enhancement[MeSH] OR Telemedicine[MeSH] OR Digital Health[MeSH] OR Mobile Applications[MeSH] OR Wearable Electronic Device[MeSH] OR Self-Help Device[Title/Abstract] OR Self Help Devices[Title/Abstract] OR Assistive Devices[Title/Abstract] OR Assistive Device[Title/Abstract] OR Assistive Technology[Title/Abstract] OR Assistive Technologies[Title/Abstract] OR Enhancement Technologies[Title/Abstract] OR Virtual Medicine[Title/Abstract] OR Mobile Health[Title/Abstract] OR mHealth[Title/Abstract] OR Telehealth[Title/Abstract] OR eHealth[Title/Abstract] OR Digital Health Technology[Title/Abstract] OR Digital Health Technologies[Title/Abstract] OR Mobile Application[Title/Abstract] OR Mobile App[Title/Abstract] OR Portable Electronic App[Title/Abstract] OR Portable Electronic Applications[Title/Abstract] OR Portable Electronic Application[Title/Abstract] OR Portable Software Apps[Title/Abstract] OR Portable Software Applications[Title/Abstract] OR Smartphone Apps[Title/Abstract] OR Smartphone App[Title/Abstract] OR Wearable Devices[Title/Abstract] OR Wearable Device[Title/Abstract] OR Wearable Technology[Title/Abstract] OR Wearable Technologies[Title/Abstract] OR Electronic Skin[Title/Abstract] OR Wearable Computer[Title/Abstract] OR Wearable Computers[Title/Abstract]) AND (Metabolic Syndrome[MeSH] OR Blood Pressure[MeSH] OR Triglycerides[MeSH] OR "Cholesterol, HDL"[MeSH] OR Blood Glucose[MeSH] OR Insulin[MeSH] OR Body Weight[MeSH] OR Waist-Hip Ratio[MeSH] OR Waist Circumference[MeSH] OR Body Weights[Title/Abstract] OR Blood Sugar[Title/Abstract] OR High Density Lipoprotein Cholesterol[Title/Abstract] OR Metabolic Syndromes[Title/Abstract] OR Reaven Syndrome X[Title/Abstract] OR Metabolic Syndrome X[Title/Abstract] OR Insulin Resistance Syndrome X[Title/Abstract] OR Metabolic Cardiovascular Syndrome[Title/Abstract] OR Metabolic X Syndrome[Title/Abstract] OR Dysmetabolic Syndrome X[Title/Abstract] OR Cardiometabolic Syndrome[Title/Abstract] OR Cardiometabolic Syndromes[Title/Abstract])) AND (Adolescent[MeSH] OR Young Adults[MeSH] OR Adolescence[Title/Abstract] OR Female Adolescent[Title/Abstract] OR Male Adolescent[Title/Abstract] OR Youth[Title/Abstract] OR Youths[Title/Abstract] OR Teens[Title/Abstract] OR Teen[Title/Abstract] OR Teenagers[Title/Abstract] OR Teenager[Title/Abstract] OR college student[Title/Abstract] OR undergraduate[Title/Abstract])

Medline

(((#1) AND(#4)) OR ((#2) AND (#3))) AND (#5)

(((TS=(Wearable Electronic Device *OR* Wearable Devices *OR* Wearable Device *OR* Wearable Technology *OR* Wearable Technologies *OR* Electronic Skin *OR* Wearable Computer *OR* Wearable Computers)) AND (TS=(Health Behavior *OR* Health-Related Behavior *OR* Health-Seeking Behavior *OR* Health-Seeking Behaviors *OR* Health-Promoting Behaviors *OR* Physical Activity *OR* Exercise *OR* Sedentary Behavior *OR* Energy Intake *OR* Overnutrition *OR* Ketogenic Diet *OR* Physical Exertion))) OR ((TS=(Wearable Armband OR Heart Rate Monitor OR Fitness Tracker OR Smartwatch OR Activity Tracker OR Smart Band OR Wearable Watch OR Wearable ECG Monitor OR Wearable Blood Pressure Monitor OR Sleep Tracker OR Wearable Pulse Oximeter OR Smart Clothing OR Smart Footwear OR Wearable Sensors OR Wearable Thermometer OR Wearable Health Monitoring Device OR Wearable Health Tracker OR Wearable ECG Patch OR Wearable Glucose Monitor OR Wearable Blood Pressure Cuff OR Wearable Thermometer OR Wearable ECG Patch OR Wearable Glucose Monitor OR Pedometer OR Electronic Activity Monitor System OR Global Positioning System OR Smart Ring OR Smart Earbud OR Medical Earbud OR Biosensor Patch OR Smart Patch OR Smart Inhaler OR Smart Footwear OR Wearable Camera OR Wearable UV Sensor OR Wearable Electrolyte Monitor OR Wearable Electrocardiogram OR Wearable Respiratory Monitor OR Wearable Sleep Apnea Monitor OR Wearable Hydration Monitor OR Wearable Stress Monitor OR Wearable Posture Corrector OR Wearable Calorie Counter OR Wearable Glucose Patch OR Wearable Blood Oxygen Monitor OR Wearable Skin Temperature Sensor OR Wearable Activity Monitor OR Wearable Fitness Band OR Wearable Wellness Tracker OR Wearable Health Sensor OR Wearable Performance Monitor OR Wearable Biofeedback Device OR Wearable Health Tracker OR Wearable Fitness Tracker OR Wearable Wellness Monitor OR Wearable Health Monitoring System OR Wearable Activity Monitoring System OR Wearable Health Tracking System OR Wearable Fitness Monitoring System OR Wearable Wellness Tracking System OR Wearable Health Monitoring Platform OR Wearable Fitness Monitoring Platform OR Wearable Wellness Monitoring Platform OR Wearable Health Tracking Platform OR Wearable Fitness Tracking Platform OR Wearable Wellness Tracking Platform OR Wearable Food Tracker OR Smart Food Recognition Device OR Smart Food Scale OR Wearable Calorie Counter OR Wearable Diet Monitoring Device OR Smart Plate OR Wearable Nutrition Monitor OR Smart Drink Monitor OR Wearable Food Logging Device OR Smart Eating Sensor)) AND (TS=(Obesity management *OR* Obesity Treatment *OR* Obesity Prevention *OR* Obesity Reduction)))) AND (TS=(Adolescent *OR* Adolescence *OR* Female Adolescent *OR* Male Adolescent *OR* Youth *OR* Youths *OR* Teens *OR* Teen *OR* Teenagers *OR* Teenager *OR* Young Adult *OR* Young Adults *OR* college student *OR* undergraduate))

Embase

(((#1) AND(#4)) OR ((#2) AND (#3))) AND (#5)

(('wearable electronic device'/exp OR 'wearable electronic device':ti,ab,kw OR 'wearable device'/exp OR 'wearable device':ti,ab,kw OR 'wearable technology'/exp OR 'wearable technology':ti,ab,kw OR 'electronic skin'/exp OR 'electronic skin':ti,ab,kw OR 'wearable computer'/exp OR 'wearable computer':ti,ab,kw OR 'wearable devices':ti,ab,kw OR 'wearable technologies':ti,ab,kw OR 'wearable computers':ti,ab,kw) AND ('health behavior'/exp OR 'health behavior':ti,ab,kw OR 'physical activity'/exp OR 'physical activity':ti,ab,kw OR 'sedentary lifestyle'/exp OR 'sedentary lifestyle':ti,ab,kw OR 'caloric intake'/exp OR 'caloric intake':ti,ab,kw OR 'overnutrition'/exp OR 'overnutrition':ti,ab,kw OR 'ketogenic diet'/exp OR 'ketogenic diet':ti,ab,kw OR 'exercise'/exp OR 'exercise':ti,ab,kw OR 'health-related behavior':ti,ab,kw OR 'health-seeking behavior':ti,ab,kw OR 'health-seeking behaviors':ti,ab,kw OR 'health-promoting behaviors':ti,ab,kw OR 'sedentary behavior':ti,ab,kw OR 'energy intake':ti,ab,kw OR 'physical exertion':ti,ab,kw) OR (('obesity management':ti,ab,kw OR 'obesity treatment':ti,ab,kw OR 'obesity prevention':ti,ab,kw OR 'obesity reduction':ti,ab,kw) AND ('fitness tracker'/exp OR 'fitness tracker':ti,ab,kw OR 'activity tracker'/exp OR 'activity tracker':ti,ab,kw OR 'wearable blood pressure monitor'/exp OR 'wearable blood pressure monitor':ti,ab,kw OR 'wearable sleep tracker'/exp OR 'wearable sleep tracker':ti,ab,kw OR 'smart clothing'/exp OR 'smart clothing':ti,ab,kw OR 'pedometer'/exp OR 'pedometer':ti,ab,kw OR 'global positioning system'/exp OR 'global positioning system':ti,ab,kw OR 'smart ring'/exp OR 'smart ring':ti,ab,kw OR 'wearable camera'/exp OR 'wearable camera':ti,ab,kw OR 'wearable armband':ti,ab,kw OR 'heart rate monitor':ti,ab,kw OR 'smartwatch':ti,ab,kw OR 'smart band':ti,ab,kw OR 'wearable watch':ti,ab,kw OR 'wearable ecg monitor':ti,ab,kw OR 'sleep tracker':ti,ab,kw OR 'wearable pulse oximeter':ti,ab,kw OR 'wearable sensors':ti,ab,kw OR 'wearable health monitoring device':ti,ab,kw OR 'wearable blood pressure cuff':ti,ab,kw OR 'wearable thermometer':ti,ab,kw OR 'wearable ecg patch':ti,ab,kw OR 'wearable glucose monitor':ti,ab,kw OR 'electronic activity monitor system':ti,ab,kw OR 'smart earbud':ti,ab,kw OR 'medical earbud':ti,ab,kw OR 'biosensor patch':ti,ab,kw OR 'smart patch':ti,ab,kw OR 'smart inhaler':ti,ab,kw OR 'smart footwear':ti,ab,kw OR 'wearable uv sensor':ti,ab,kw OR 'wearable electrolyte monitor':ti,ab,kw OR 'wearable electrocardiogram':ti,ab,kw OR 'wearable respiratory monitor':ti,ab,kw OR 'wearable sleep apnea monitor':ti,ab,kw OR 'wearable hydration monitor':ti,ab,kw OR 'wearable stress monitor':ti,ab,kw OR 'wearable posture corrector':ti,ab,kw OR 'wearable glucose patch':ti,ab,kw OR 'wearable blood oxygen monitor':ti,ab,kw OR 'wearable skin temperature sensor':ti,ab,kw OR 'wearable activity monitor':ti,ab,kw OR 'wearable fitness band':ti,ab,kw OR 'wearable wellness tracker':ti,ab,kw OR 'wearable health sensor':ti,ab,kw OR 'wearable performance monitor':ti,ab,kw OR 'wearable biofeedback device':ti,ab,kw OR 'wearable health tracker':ti,ab,kw OR 'wearable fitness tracker':ti,ab,kw OR 'wearable wellness monitor':ti,ab,kw OR 'wearable health monitoring system':ti,ab,kw OR 'wearable activity monitoring system':ti,ab,kw OR 'wearable health tracking system':ti,ab,kw OR 'wearable fitness monitoring system':ti,ab,kw OR 'wearable wellness tracking system':ti,ab,kw OR 'wearable health monitoring platform':ti,ab,kw OR 'wearable fitness monitoring platform':ti,ab,kw OR 'wearable wellness

monitoring platform':ti,ab,kw OR 'wearable health tracking platform':ti,ab,kw OR 'wearable fitness tracking platform':ti,ab,kw OR 'wearable wellness tracking platform':ti,ab,kw OR 'wearable food tracker':ti,ab,kw OR 'smart food recognition device':ti,ab,kw OR 'smart food scale':ti,ab,kw OR 'wearable calorie counter':ti,ab,kw OR 'wearable diet monitoring device':ti,ab,kw OR 'smart plate':ti,ab,kw OR 'wearable nutrition monitor':ti,ab,kw OR 'smart drink monitor':ti,ab,kw OR 'wearable food logging device':ti,ab,kw OR 'smart eating sensor':ti,ab,kw))) AND ('adolescent':ti,ab,kw OR 'adolescence behavior':ti,ab,kw OR 'female adolescent':ti,ab,kw OR 'male adolescent':ti,ab,kw OR 'youth':ti,ab,kw OR 'youths':ti,ab,kw OR 'teens':ti,ab,kw OR 'teen':ti,ab,kw OR 'teenagers':ti,ab,kw OR 'teenager':ti,ab,kw OR 'young adult':ti,ab,kw OR 'young adults':ti,ab,kw OR 'college student':ti,ab,kw OR 'undergraduatet':ti,ab,kw)

Scopus

(((#1) AND(#4)) OR ((#2) AND (#3))) AND (#5)

(((TITLE-ABS-KEY ("Wearable Electronic Device" OR "Wearable Devices" OR "Wearable Device" OR "Wearable Technology" OR "Wearable Technologies" OR "Electronic Skin" OR "Wearable Computer" OR "Wearable Computers")) AND (TITLE-ABS-KEY ("Health Behavior" OR "Health-Related Behavior" OR "Health-Seeking Behavior" OR "Health-Seeking Behaviors" OR "Health-Promoting Behaviors" OR "Physical Activity" OR "Exercise" OR "Sedentary Behavior" OR "Energy Intake" OR "Overnutrition" OR "Ketogenic Diet" OR "Physical Exertion"))) OR ((TITLE-ABS-KEY ("Obesity management" OR "Obesity Treatment" OR "Obesity Prevention" OR "Obesity Reduction")) AND (TITLE-ABS-KEY ("Wearable Armband" OR "Heart Rate Monitor" OR "Fitness Tracker" OR "Smartwatch" OR "Activity Tracker" OR "Smart Band" OR "Wearable Watch" OR "Wearable ECG Monitor" OR "Wearable Blood Pressure Monitor" OR "Sleep Tracker" OR "Wearable Pulse Oximeter" OR "Smart Clothing" OR "Smart Footwear" OR "Wearable Sensors" OR "Wearable Thermometer" OR "Wearable Health Monitoring Device" OR "Wearable Health Tracker" OR "Wearable ECG Patch" OR "Wearable Glucose Monitor" OR "Wearable Blood Pressure Cuff" OR "Pedometer" OR "Electronic Activity Monitor System" OR "Global Positioning System" OR "Smart Ring" OR "Smart Earbud" OR "Medical Earbud" OR "Biosensor Patch" OR "Smart Patch" OR "Smart Inhaler" OR "Wearable Camera" OR "Wearable UV Sensor" OR "Wearable Electrolyte Monitor" OR "Wearable Electrocardiogram" OR "Wearable Respiratory Monitor" OR "Wearable Sleep Apnea Monitor" OR "Wearable Hydration Monitor" OR "Wearable Stress Monitor" OR "Wearable Posture Corrector" OR "Wearable Calorie Counter" OR "Wearable Glucose Patch" OR "Wearable Blood Oxygen Monitor" OR "Wearable Skin Temperature Sensor" OR "Wearable Activity Monitor" OR "Wearable Fitness Band" OR "Wearable Wellness Tracker" OR "Wearable Health Sensor" OR "Wearable Performance Monitor" OR "Wearable Biofeedback Device" OR "Wearable Health Tracker" OR "Wearable Fitness Tracker" OR "Wearable Wellness Monitor" OR "Wearable Health Monitoring System" OR "Wearable Activity Monitoring System" OR "Wearable Health Tracking System" OR "Wearable Fitness Monitoring System" OR "Wearable Wellness Tracking System" OR "Wearable Health Monitoring Platform" OR "Wearable Fitness Monitoring Platform" OR "Wearable Wellness Monitoring Platform" OR "Wearable Health Tracking Platform" OR "Wearable Fitness Tracking Platform" OR "Wearable Wellness Tracking

Platform" OR "Wearable Food Tracker" OR "Smart Food Recognition Device" OR "Smart Food Scale" OR "Wearable Diet Monitoring Device" OR "Smart Plate" OR "Wearable Nutrition Monitor" OR "Smart Drink Monitor" OR "Wearable Food Logging Device" OR "Smart Eating Sensor")))) AND (TITLE-ABS-KEY ("Adolescent" OR "Adolescence" OR "Female Adolescent" OR "Male Adolescent" OR "Youth" OR "Youths" OR "Teens" OR "Teen" OR "Teenagers" OR "Teenager" OR "young adult" OR "young adults" OR "college student" OR "undergraduate"))
