# Supplementary material for: How Emerging Digital Health Technologies Based on Dietary and Physical Activity Regulation Improve Metabolic Syndrome-Related Outcomes in Adolescents: A Systematic Review
Source: Metabolites. 2026 Feb 2;16(2):106. doi: 10.3390/metabo16020106 (PMC12942388; doi:10.3390/metabo16020106)
Supplement: Supplementary file 1 [file metabolites-16-00106-s001.zip › Table S1 Detailed characteristics of the 12 studies analyze.pdf]

Detailed characteristics of the 12 studies analyzed in this research

No.	Title	Author, Year	Journal	Digital health technology	Population	Duration	Age group (average age)	Intervention group (n)	Control Group (n)	Intervention /Control Content	Outcome sample (n)	Main outcomes	Effect size
1	Preventing obesity among adolescent girls One-year outcomes of the nutrition and enjoyable activity for teen girls (NEAT girls) cluster randomized controlled trial	Lubans 2012	ARCH PEDIAT ADOL MED	Multi-component lifestyle intervention	Adolescent girls (low SES)	12 months	12–14 (13.18±0.45)	Combined intervention (n=178)	Usual PE (n=179)	**Intervention Content:** School-based program + pedometers + SMS<br>**Control Content:** usual curriculum	IG (n=141) CG (n=153)	**BMI:** The intervention group showed a downward trend, but neither within-group nor between-group differences were statistically significant. **BMI z-score:** A downward trend was observed in the intervention group, but within-group and between-group differences were not significant.	**BMI:** AMD = -0.19 （95% CI -0.70 to 0.33） **BMI z-score:** AMD = -0.08 （95% CI -0.20 to 0.04）
2	An Electronic Wellness Program to Improve Diet and Exercise in College Students A Pilot Study	Schweitzer 2016	JMIR RES PROTOC	Email-based intervention	College students	24 weeks	18–20 (19.7±0.06)	Combined intervention (n=99)	Usual guidance (n=49)	**Intervention Content:** weekly personalized diet and PA goals and feedback via email (ALIVE program), with website access and progress tracking<br>**Control Content:** weekly non-diet, non-PA health information (e.g., sleep, distracted driving)	IG (n=68) CG (n=38)	**BMI:** No significant changes were observed in either group over 24 weeks (P=0.80). **WC and WHR:** No significant between-group differences were observed (P=0.41 and P=0.21). **BP (SBP/DBP):** No significant between-group differences were observed (P=0.92 and P=0.80).	**BMI:** NR **WC and WHR:** NR **BP (SBP/DBP):** NR
3	Short-Term Efficacy of an Innovative Mobile Phone Technology-Based Intervention for	Chen 2017	INTERACT J MED RES	Wearable devices plus online courses and text message	Overweight/obese adolescents	6 months	13–18 (14.9±1.7)	Combined intervention (n=23)	Single physical activity (n=17)	**Intervention Content:** Wearable (Fitbit) + online modules + SMS	IG (n=21) CG (n=15)	**BMI:** The intervention group showed a significantly greater reduction than the control group (P=0.001).	**BMI:** Cohen’ s d = 0.62 **BMI z-score:** Cohen’s d = 0.34 **WHR:** Cohen’ s d

	Weight Management for Overweight and Obese Adolescents Pilot Study			intervention						**Control Content:** pedometer and general health modules		**BMI z-score:** The intervention group showed a significantly greater reduction than the control group (P=0.001). **WHR:** No significant change was observed (P>0.05). **BP:** Overall BP decreased significantly in the intervention group compared with the control group (P=0.001), whereas SBP showed no significant difference.	= 0.22 **BP: SBP:** Cohen' s d = 0.06 **DBP:** Cohen' s d = 0.21
4	Wearable activity tracking device use in an adolescent weight management clinic A randomized controlled pilot trial	Bowen-Jallow 2021	J OBES	Wearable device (Fitbit)	Obese adolescents (clinic-base)	18 weeks	12–18 (14.5±1.7)	Physical activity intervention (n=18)	Usual care (n=30)	**Intervention Content:** Wearable activity tracker **Control Content:** Usual care	IG (n=10) CG (n=23)	**BMI:** No significant between-group difference was observed (P=1.00). **WC:** No significant between-group difference was observed (P=0.83).	**BMI:** SD = 9.39 **WC:** SD = 22.8
5	A randomized trial comparing diet and delivery strategies for weight management in adolescents with intellectual disabilities	Ptomey 2023	PEDIATR OBES	Remote video sessions + digital self-monitoring (Fitbit and mobile application)	Adolescents with intellectual disabilities and overweight/obesity	18 months	13–21 (16)	Combined intervention (n=36)	Usual diet (n=74)	**Intervention Content:** Telehealth + enhanced Stop-Light Diet + PA self-monitoring **Control Content:** remote video/face-to-face visits + conventional diet	IG (n=30) CG (n=58)	**BMI:** The intervention group showed a significantly greater reduction than the control group (P=0.03). **WC:** The intervention group showed a significantly greater reduction than the control group (P=0.002).	**BMI:** MD = -1.5 **WC:** MD = -4.7
6	Use of activity trackers to improve blood pressure in young people at risk for cardiovascular disease A pilot	Bicki 2024	PEDIATR NEPHROL	Wearable device (Fitbit) + email or telephone contact	Youth at cardiovascular risk	6 months	8–30 (18±4)	Physical activity intervention (n=42)	Usual care (n=21)	**Intervention Content:** Activity tracker + biweekly feedback **Control Content:**	IG (n=26) CG (n=18)	**SBP:** Over 6 months, SBP decreased by -2.3 mmHg in the intervention group (95%CI, -6.5 to 1.8), with no significant between-group difference	**SBP:** -2.3 mmHg (95% CI -6.5, 1.8)

	randomized controlled trial									Usual care		(P=0.12).	
7	A mobile app-based intervention improves anthropometry, body composition and fitness, regardless of previous active-inactive status a randomized controlled trial	Gómez-Cuesta 2024	FRONT PUBLIC HEALTH	Step tracking application	Secondary school adolescents	10 weeks	12–16 (13.76±1.41)	Physical activity intervention (n=280)	Usual PE (n=182)	**Intervention Content:** Step tracker mobile apps (Pokémon Go, Strava, Pacer) integrated into PE **Control Content:** No step-tracking app usual school PE only	IG (n=270) CG (n=160)	**BMI:** The intervention group showed no significant change (P=0.344), the control group showed a significant change (P=0.001) **WHtR:** No significant changes were observed in either group (P=0.129 and P=0.187).	**BMI:** η² = 0.002, 0.027 **WHtR:** η²= 0.006, 0.005
8	Physical activity, body composition, and fitness variables in adolescents after periods of mandatory, promoted or nonmandatory, nonpromoted use of step tracker mobile apps Randomized controlled trial	Mateo-Orcajada 2024	JMIR MHEALTH UHEALTH	Step tracking application	General adolescent population	20 weeks	12–16 (13.92±1.91)	Physical activity intervention (n=300)	Usual PE (n=165)	**Intervention Content:** Mandatory step tracking application use **Control Content:** Usual school PE only	IG (n=216) CG (n=141)	**BMI and WC:**No significant between-group differences were observed at any time point (P=0.09, P=0.61). **WHR:** The intervention group showed a significantly greater reduction than the control group (P=0.02).	**BMI:** ηp²= 0.009 **WC:** ηp²= 0.001 **WHR:** ηp²= 0.017
9	Effect of eight-week high-intensity interval training versus moderate-intensity continuous training programme on body composition, cardiometabolic risk factors in sedentary adolescents	Sun 2024	FRONT PHYSIOL	Wearable metabolic system (K5 Wearable Metabolic System) plus heart rate monitor (Polar OH1 Model 2L)	Sedentary adolescents	8 weeks	~18 (18.5±0.3)	Physical activity intervention (n=6)	MICT (n=6)	**Intervention Content:** Training is monitored throughout via wearable devices, with heart rate maintained between 85% and 95% of maximum heart rate. **Control Content:**	IG (n=6) CG (n=6)	**BMI:** No significant change was observed (P>0.05). **WHR:** A significant reduction was reported in the intervention group (P=0.033). **VFA:** Both the intervention and control groups decreased significantly respectively (P=0.001 and P=0.003). **BP:** SBP and DBP decreased significantly in	**BMI:** NR **WHR:** ES = 0.43 **VFA:** ES = 0.35, 0.49 **BP:** ES = 0.84, 1.76 **TG:** ES = 1.33 **BG:** NR

										Training is monitored throughout via wearable devices, with heart rate maintained between 65% and 75% of maximum heart rate.		the intervention group respectively (P=0.018 and P=0.008).<br>**BL:** TG decreased significantly in the intervention group (P=0.004), whereas TC, HDL-C, and LDL-C showed no significant changes (P>0.05).<br>**BG:** No significant between-group difference was observed for BG, and HOMA-IR showed a non-significant downward trend in the intervention group.	
10	The impact of behavior change counseling delivered via a digital health tool versus routine care among adolescents with obesity Pilot randomized feasibility study	Kepper 2024	JMIR formative research	Digital counseling tool (PREVENT)	Adolescents with obesity	3 months	12–18 (14.72± 1.85)	Combined intervention (n=18)	Usual care (n=18)	**Intervention Content:** Text messaging for dietary guidance + physical activity monitoring + integrating electronic health records + cardiovascular health visualisation (PREVENT)<br><br>**Control Content:** Usual care	IG (n=10) CG (n=15)	**BMI z-score：** Both groups showed downward trends, but changes were not statistically significant (95% CI -0.31, 0.20 and 95% CI -0.18, 0.10).<br>**BP:** SBP decreased significantly in the intervention group (within-group P=0.009; between-group P=0.001). DBP decreased significantly within the intervention group (within-group P=0.009), but the between-group difference was not significant.<br>**BG and TC:** Downward trends were reported; however, due to high missingness, statistical significance testing was not performed.(95% CI -36.83, 20.08 and 95% CI -21.46,	**BMI z-score:**<br>IG: (95% CI -0.31, 0.20),<br>CG: (95% CI -0.18, 0.10)<br>**SBP:**<br>IG: (95% CI -18.46, -2.98),<br>CG: (95% CI -5.06, 15.17)<br>**DBP:**<br>IG: (95% CI -12.86, -2.19)<br>CG: (95% CI -12.07, 2.68)<br>**BG:**<br>IG: (95% CI -36.83, 20.08),<br>CG: (95% CI -21.46, 21.29)<br>**TC:**<br>IG: (95% CI -366.45, 389.27),<br>CG: (95% CI

												21.29, 95% CI -366.45, 389.27 and 95% CI -46.92, 58.32)	-46.92, 58.32)
11	Effects of dietary advice, aerobic exercise, and virtual reality games on the quality of life of obese adolescent females	Abd El-Khalek 2025	SPORT TK - EuroAmerican Journal of Sport Sciences	Virtual reality (VR)	Obese adolescent females	8 weeks	12–17 (13.44±1.29)	Combined intervention (n=50)	No VR equipment (n=50)	**Intervention Content:** Diet + aerobic exercise + VR games<br>**Control Content:** Diet + aerobic exercise	IG (n=50) CG (n=50)	**BMI:** Both groups decreased significantly post-intervention (within-group P<0.001), and the reduction was significantly greater in the intervention group (between-group P=0.001). **WC and WHR:** Both groups improved significantly (within-group P<0.001), but between-group differences were not significant.	**BMI:** Cohen' s d = 1.73 **WC:** Cohen' s d = 0.23 **WHR:** Cohen' s d = 0.32
12	Feasibility and Effectiveness of a Social Network-Based Intervention for Adolescents Undergoing Weight Loss Treatment A Randomized Controlled Trial	Ramalho 2025	Nutrients	Social network (Facebook) + web-based self-monitoring	Overweight/obese adolescents	6 months	13–18 (14.9)	Combined intervention (n=69)	Usual guidance (n=66)	**Intervention Content:** Social-network CBT platform + self-monitoring<br>**Control Content:** Usual hospital-based lifestyle guidance	IG (n=38) CG (n=39)	**BMI z-score:** Both groups decreased significantly over time (P=0.006), with no significant group-by-time interaction.	**BMI z-score:** ηp^2= 0.116

Note: **BMI**: Body mass index; BMI z-score; **WC:** Waist circumference; **WHtR:** Waist-to-height ratio; **WHR:** Waist-to-hip ratio; **VFA:** Visceral fat area; **BP:** Blood pressure; **BG**: Blood glucose; **HOMA-IR:** Homeostatic model assessment of insulin resistance ; **TG:** Triglycerides; **TC:** Total cholesterol; **HDL-C:** High-density lipoprotein cholesterol; **LDL-C:** Low-density lipoprotein cholesterol; **ARCH PEDIAT ADOL MED:** Archives of pediatrics & adolescent medicine; **JMIR RES PROTOC:** JMIR Research Protocols; **INTERACT J MED RES:** Interactive Journal of Medical Research; **J OBES:** Journal of Obesity; PEDIATR OBES: ; **PEDIATR NEPHROL:** Pediatric Obesity; **JMIR MHEALTH UHEALTH:** JMIR mHealth and uHealth; **FRONT PHYSIOL:** Frontiers in Physiology.
