# Supplementary material for: How Emerging Digital Health Technologies Based on Dietary and Physical Activity Regulation Improve Metabolic Syndrome-Related Outcomes in Adolescents: A Systematic Review
Source: Metabolites. 2026 Feb 2;16(2):106. doi: 10.3390/metabo16020106 (PMC12942388; doi:10.3390/metabo16020106)
Supplement: Supplementary file 1 [file metabolites-16-00106-s001.zip › Table S2 Concise version Characteristics and outcomes included in the study.pdf]

Concise version: Characteristics and outcomes included in the study

Study	Country	Design	Population	Age (years)	Intervention (Digital Component)	Duration	Control	Key Metabolic Outcomes
Lubans et al., 2012	Australia	Cluster RCT	Adolescent girls (low SES)	12–14	School-based program + pedometers + SMS	12 months	Usual PE	**BMI** [1, 6, 7]<br>**BMI z-score** [1, 6, 7]
Schweitzer et al., 2016	USA	RCT (pilot)	College students	18–20	Email-based eHealth lifestyle coaching	24 weeks	Usual guidance	**BMI** [5, 6]<br>**WC and WHR** [5, 6]<br>**BP** (SBP/DBP) [5, 6]
Chen et al., 2017	USA	RCT (pilot)	Overweight/obese adolescents	13–18	Wearable (Fitbit) + online modules + SMS	6 months	Single physical activity	**BMI** [2]<br>**BMI z-score** [2]<br>**WHR** [5, 6]<br>**BP:** Overall BP [2], SBP [1]
Bowen-Jallow et al., 2021	USA	RCT (pilot)	Obese adolescents (clinic-based)	12–18	Wearable tracker adjunct to clinic	18 weeks	Usual care	**BMI** [1]<br>**WC** [1]
Ptomey et al., 2023	USA	RCT	Adolescents with intellectual disabilities, overweight/obese	13–21	Telehealth + enhanced Stop-Light Diet + PA self-monitoring	18 months	Usual diet	**BMI** [2]<br>**WC** [2]
Bicki et al., 2024	USA	RCT (pilot)	Youth at cardiovascular risk	8–30	Activity tracker + biweekly feedback	6 months	Usual care	**SBP** [1, 7]
Gómez-Cuesta et al., 2024	Spain	RCT	Secondary school adolescents	12–16	Step-tracker mobile apps (Pokémon Go, Strava, Pacer) integrated into PE	10 weeks	Usual PE	**BMI** [3]<br>**WHtR** [1, 3, 4]
Mateo-Orcajada et al., 2024	Spain	RCT	General adolescent population	12–16	Mandatory vs voluntary step-tracker use	20 weeks	Usual PE	**BMI** [1]<br>**WC** [1]<br>**WHR** [2]
Sun et al., 2024	China	RCT	Sedentary adolescents	~18	HIIT vs MICT (Wearable metabolic system)	8 weeks	MICT	**BMI** [1, 5, 6]<br>**WHR** [3]<br>**VFA** [3, 4]<br>**BP** [3]<br>**BL:** TG [3] , (TC, HDL-C, and LDL-C)[1, 3, 4]<br>**BG:** BG [1, 3, 4], HOMA-IR [7]
Kepper et al., 2024	USA	RCT (pilot)	Adolescents with obesity	12–18	Clinician-delivered digital counseling tool (PREVENT)	3 months	Usual care	**BMI z-score** [8]<br>**BP:** SBP [2, 3], DBP [3]<br>**TC** [8]<br>**BG** [8]
Abd El-Khalek et al., 2025	Egypt	RCT	Obese adolescent females	12–17	Diet + aerobic exercise + VR games	8 weeks	No VR equipment	**BMI** [2, 3, 4]<br>**WC and WHR** [1, 3, 4]
Ramalho et al., 2025	Portugal	RCT	Overweight/obese adolescents	13–18	Social-network CBT platform + self-monitoring	6 months	Usual guidance	**BMI z-score** [3, 4]

Note : 1. No significant difference between groups; 2. Significant differences between groups; 3. Significant changes within the intervention group; 4. Significant changes within the control group; 5. No significant changes occurred within the intervention group; 6. No significant changes occurred within the intervention group; 7. Intervention group showed a downward trend but this was not statistically significant; 8. Two groups showed a downward trend but this was not statistically significant.
